# Pro-efferocytic macrophage membrane biomimetic nanoparticles for the synergistic treatment of atherosclerosis via competition effect

**DOI:** 10.1186/s12951-022-01720-2

**Published:** 2022-12-01

**Authors:** Xuan Sha, Yue Dai, Lijuan Chong, Min Wei, Mengyuan Xing, Chun Zhang, Jingjing Li

**Affiliations:** 1 School of Medical Imaging, Xuzhou Medical University, Xuzhou, 221004 China; 2 Department of Radiology, Affiliated Hospital of Xuzhou Medical University, Xuzhou, 221006 China

**Keywords:** Macrophage membrane, Biomimetic nanoparticles, Efferocytosis, Atherosclerosis, Synergistic treatment

## Abstract

Macrophages participate in many links in the pathological process of atherosclerosis (AS) and the regulation of influence of macrophages at the molecular level might be a new avenue for AS treatment. For this aim, the macrophage membrane biomimetic nanoparticles, derived from macrophage membrane coated SHP1i-loaded liposome NPs (MM@Lips-SHP1i) was designed. Due to the reservation of intrinsic membrane proteins and function from macrophages, the biomimic nanoparticles could effectively evade clearance by the immune system, prolong blood circulation time and actively tend and aggregate to atherosclerotic plaques. More importantly, in the plaque area, MM@Lips-SHP1i nanoparticles could compete with macrophages *in vivo* to bind with oxidized low-density lipoprotein (oxLDL) and lipopolysaccharide (LPS), reduce uptake of new lipids by macrophages, reduce foam cell formation, and inhibit the expression of pro-inflammatory cytokines. In addition, small molecule inhibitor of SHP-1, the downstream effector molecule of CD47 loaded in macrophage membrane biomimetic nanoparticles could interrupt CD47-SIRPα signal transduction in monocytes and macrophages, thereby enhancing the efferocytosis of macrophages, inhibiting the progression of plaque, achieving synergistic treatment of atherosclerosis. This work focuses on the key process in the formation of AS, macrophage foaming and chronic inflammation, and is based on the fact that macrophage membrane biomimetic nanoparticles can preserve the key surface proteins of macrophages closely related to the formation of AS, providing a new avenue to inhibit the progression of AS by utilizing the biological characteristics of macrophage membrane in macrophage membrane biomimetic nanoparticles.

**Supplementary Information:**

The online version contains supplementary material available at 10.1186/s12951-022-01720-2.

## Background

The morbidity and mortality of cardiovascular disease are increasing year by year globally, and about 17.3 million people die of this disease every year [1]. Atherosclerosis (AS) plaque instability and rupture are the main cause of death for patients with cardiovascular diseases in China. AS is a chronic progressive vascular inflammatory disease [2]. The understanding of AS progress at the molecular level shows that cholesterol and lipid deposition and chronic inflammation play a key role in the occurrence and development of AS [3]. Macrophages participate in many links in the pathological process of AS, such as promoting the plaque formation, diluting fiber cap and necrotic core components, and leading to thrombosis and plaque disintegration [4, 5]. Foam cells in the early AS lesions mainly come from macrophages. Macrophages uptake oxLDL to accumulate lipids, resulting in the formation of foam cells, followed releasing a variety of enzymes and inflammatory mediators, which further accelerates the progression of AS plaque and lesions [6, 7]. The key role of macrophages in the development of AS inspired us to explore the possibility of macrophage membrane biomimetic nanoparticles using the biological characteristics of the membrane to inhibit the progression of AS.

Biomimetic nanoparticles coated with cell membrane have been widely developed due to their advantages of high biocompatibility, strong targeting and immune escape ability [8]. They can mimic the different functions of primary cells while successfully delivering loaded drugs, thereby possessing the potential to improve the limited efficacy of drugs in clinics resulting from the rapid drug clearance and unsatisfactory accumulation at the arterial injury site [9]. For example, Ge et al. reported platelet membrane-coated nanoparticle-mediated targeting delivery of rapamycin with a 4.98-fold greater efficiency than that without platelet membrane coating in the anti-atherosclerosis effect [10]. Similar biomimetic nanoparticles fabricated with macrophage membrane or red blood cell membrane coating on the surface of rapamycin-loaded poly(lactic-co-glycolic acid) copolymer (PLGA) nanoparticles also displayed the effectively accumulation in atherosclerotic lesions in vivo and significantly delayed the progression of AS [2, 11]. Different from other cells in the progression of AS, macrophages not only have the ability to cross physiological barriers, escape immune recognition, intracellular transport, target inflamed sites, but also aggravate the AS progression by uptaking a large amount of oxLDL to form foam cells and releasing the proinflammatory cytokines including tumor necrosis factor-α (TNF-α), and interleukin-6 (IL-6) to recruit monocytes [12]. Considering such dual function of macrophage, in this study, we developed macrophage membrane biomimetic nanoparticles derived from macrophage membrane coated liposome NPs (MM@Lips NPs) to explore their inhibition ability on AS progression by the biological characteristics of the membrane. The MM@Lips NPs kept the same antigenic appearance as the source macrophages, and have the inherent ability to homing to inflammation and evade the body’s defense mechanisms. Meanwhile, the preserve of scavenger receptors such as SRA I/II, SRB I, CD36, Toll-like receptors (TLRs) and other important proteins guaranteed their ability to bind to oxLDL and LPS, providing the basis for the treatment of atherosclerosis through the biological characteristics of macrophage membrane.

In addition, recent studies have found that defective efferocytosis of macrophages is also a key factor in the progression of AS plaques. The expansion of necrotic core within the plaques is not due to the existence of too many dead cells, but due to the little clearance of dead cells [13]. Efferocytosis refers to the process of phagocytic cells engulfing and degrading senescent, damaged or dead cells, which can prevent secondary necrosis and inflammation [14]. About 10^6^ apoptotic or senescent cells are “processed” every second in the human body, but in AS plaques, the efferocytosis is only about 1/20 of the physiological level, which partly comes from the recognition of apoptotic cells by macrophage or macrophage's own dysfunction [13]. Therefore, activating or reactivating the elimination of apoptotic cells in plaques to improve plaque stability and even promote plaque regression may also be an important avenue for the prevention and treatment of AS-related diseases.

Thus, to further improve the treatment effect of macrophage biomimetic nanoparticles, a SHP1 inhibitor (SHP1i) was loaded in liposome. SHP-1 is a small-molecule inhibitor of CD47’s downstream effector molecule. The upregulation of CD47, a ‘don’t eat me’ molecule, is a major mechanism to deliver an antiphagocytic signal. In AS plaque, the upregulated CD47 binds with the signal regulatory protein-α (SIRPα) on macrophages to activate the Src homology 2 domain-containing phosphatase-1 (SHP-1), mediating the intracellular signaling suppressesion of phagocytic function [15, 16]. The blocking of CD47-SIRPα signaling pathway can enhance the clearance of apoptotic cells by macropahges and stimulate cell proliferation, reduce cholesterol accumulation, promote lipid efflux, attenuate oxLDL-induced inflammation, induce M2-type macrophages and inhibit the formation of necrotic core in the arterial wall [17, 18].

The ability of the final nanoformulation, MM@Lips-SHP1i to escape from immune system, accumulate in AS plaques, reduce the formation of foaming cells, inhibit the expression of pro-inflammatory cytokines and ROS, enhance the efferocytosis of macrophages and eventually inhibit the progression of AS plaques was evaluated both *in vitro* and *in vivo*.

## Materials and methods

### Materials

1,2-Distearoyl-sn-glycero-3-phosphocholine (DSPC), 1,2-distearoyl-sn-glycero-3-phosphoethanolamine-N-[methoxy(polyethylene glycol)-2000] (DSPE-mPEG2000) and DSPE-mPEG2000-COOH were purchased from Xi'an Ruixi Biological Technology Co., Ltd. (Xi’an, China). Cholesterol was purchased from Shanghai Aladdin Biochemical Technology Co., Ltd. (Shanghai, China). SHP1i were achieved from Sigma-Aldrich (USA). CD36 Polyclonal Antibody and CD130 Polyclonal Antibody was purchased from Beijing Biodragon Immunotechnologies Co., Ltd. (Beijing, China). TLR4 Polyclonal Antibody, IL-6R Polyclonal Antibody, TNFR1 Polyclonal Antibody, IFNGR1 Polyclonal Antibody, SRA Polyclonal Antibody, CD80 Polyclonal Antibody and CD206 Polyclonal Antibody were purchased from Proteintech Group, Inc (Wuhan; China). Oxidized Low Density Lipoprotein (oxLDL) was obtained from Shanghai Yuanye Bio-Technology Co., Ltd. (Shanghai, China). Lipopolysaccharide (LPS) was supplied by Sigma-Aldrich (USA). oxLDL ELISA kit was received from Jiangsu Mei Biao Biological Technology Co., Ltd. (Yancheng, China). Limulus amebocyte lysate (LAL) assay was purchased from Thermo Fisher Scientific (USA). Coumarin-6 and Oil Red O solution were purchased from Beijing Solarbio Science & Technology Co., Ltd. (Beijing, China). Total cholesterol (TC) determination kit was received from Shanghai Cablebridge Biotechnology Co., Ltd. (Shanghai, China). Mouse Tumor Necrosis Factor Alpha ELISA kit was obtained from ABclonal Biotechnology Co., Ltd. (Wuhan; China). Mouse IL-6 ELISA kit and Mouse IFN-γ ELISA kit were purchased from Beijing Solarbio Science & Technology Co., Ltd. (Beijing, China). 2′,7′- Dichlorodihydrofluorescein diacetate (DCFH-DA) was purchased from Dalian Meilun Biotechnology Co., Ltd. (Dalian, China). Annexin V-FITC Apoptosis Detection Kit was purchased from Jiangsu KeyGEN BioTECH Co., Ltd. (Nanjing, China). Cell-Tracker Red CMTPX and Cell-Tracker Green CMTPX were purchased from Shanghai Maokang Biotechnology Co., Ltd. (Shanghai, China). CD68 Polyclonal Antibody, CD14 Polyclonal Antibody, MMP-9 Polyclonal Antibody, α-SMA Polyclonal Antibody, CD31 Polyclonal Antibody, KI67 Polyclonal Antibody and Caspase-3 Polyclonal Antibody were purchased from Wuhan Servicebio Technology Co., Ltd. (Wuhan, China). Anti-Macrophage Inflammatory protein 3 alpha antibody was obtained from Abcam Plc (Shanghai, China). Rabbit Anti-phospho-PTPN6 antibody was purchased from Beijing Biosynthesis Biotechnology Co., Ltd. (Beijing, China).

### Isolation and verification of macrophage membrane

Macrophage membranes were acquired with a previously reported method, with a minor modification [19] [20]. Specifically, RAW264.7 macrophage cells (cell number ≈ 1 × 10^8^) were isolated from culture dish with 0.25% Trypsin–EDTA and washed with PBS for 3 times (500 × g for 10 min each time), and dispersed in PBS. Next, a hypotonic lysing buffer containing 1 mmol L^−1^ NaHCO_3_, 0.2 mmol L^−1^ EDTA and 1 mmol L^−1^ PMSF, was added to disperse cells and incubated in 4 °C overnight. Then, the cell suspension was put into a non-contact automatic ultrasonic disruptor and was destroyed after 60 cycles. The obtained suspension was centrifuged at 3200 × g at 4 °C for 5 min to remove large debris. The collected supernatant was further centrifuged at 20,000 × g for 25 min to discard the pellet. Finally, the supernatant was centrifuged at 100,000 × g for 35 min to collect the cell membranes in the bottom, which were dispersed in PBS (pH = 7.4). To obtain macrophage membrane vesicles, the extracted macrophage membranes were extruded 15–20 times through a 400 nm and 200 nm polycarbonate porous membrane using an Avestin Mini-extruder. The harvested macrophage membrane vesicles were stored in PBS at 4 °C for later use.

In order to verify the successful isolation of macrophage membrane, the cultured macrophages were double-stained with DiI and DAPI to distinguish the isolated membrane components from the nuclear components. Briefly, RAW264.7 cells were fixed with 4% paraformaldehyde for 15 min and washed with PBS for 3 times. Then, the cells were stained with DAPI for 3–5 min and washed with PBS for 3 times. Next, the cells were stained with 10 µM DiI for 15–20 min and washed with PBS for 3 times. The macrophage membrane was then extracted according to the above steps. The extract in each step was observed under the fluorescence microscope and photographed.

### Synthesis of blank liposomes and lips-SHPIi nanoparticles

Blank liposomes were prepared by lipid film hydration and extrusion method, according to the article we previously published with minor modification [21]. Briefly, 0.0198 g DSPC, 0.0048 g cholesterol, 0.001875 g DSPE-mPEG2000 and 0.001875 g DSPE-mPEG2000-COOH were dissolved in a mixture of chloroform (4 mL) and methanol (1 mL). The above solution was sonicated with an ultrasonic cell disruptor for 30 min. Then, the organic solvent was evaporated to dryness using a vacuum rotary evaporator, to obtain a lipid film attached to the bottle wall. Then it was placed in a vacuum drying oven to dry for 6 h. The obtained thin film was hydrated by vortexing in a 5 mL PBS solution and stirred to form blank liposomes suspension. Finally, the blank liposomes suspension can be extruded repeatedly 10 times through a 400 nm and 200 nm polycarbonate porous membrane in a liposome extruder to get well size-distributed blank liposomes for further use.

For SHP1i loading, the SHP1i solution was added to blank liposomes overnight to form Lips-SHP1i nanoparticles. After 24 h of stirring, Lips-SHP1i was centrifuged to remove unloaded SHP1i molecules. The concentration of the loaded SHP1i was measured using a NanoDrop (Nanodrop2000; Thermo Scientific) according to its absorption of 320 nm [18]. The SHP1i loading efficiency (LE) and encapsulation efficiency (EE) was determined by the following equations:

$$\mathrm{Loading\,Efficiency }\,(\mathrm{LE})=\frac{{\mathrm{m}}_{\mathrm{SHP}1\mathrm{i},\mathrm{total}}-{\mathrm{m}}_{\mathrm{SHP}1\mathrm{i},\mathrm{free}}}{{\mathrm{m}}_{\mathrm{blank liposomes}}}\times 100\mathrm{\%}$$

$$\mathrm{Encapsulation\,Efficiency }\,(\mathrm{EE})=\frac{{\mathrm{m}}_{\mathrm{SHP}1\mathrm{i},\mathrm{total}}-{\mathrm{m}}_{\mathrm{SHP}1\mathrm{i},\mathrm{free}}}{{\mathrm{m}}_{\mathrm{SHP}1\mathrm{i},\mathrm{total}}}\times 100\mathrm{\%}$$

### Preparation and characterization of MM@Lips and MM@Lips-SHP1i

A mechanical co-extrusion method was used to fuse the macrophage membrane and the blank liposomes or Lips-SHP1i nanoparticles to obtain the macrophage membrane biomimetic nanoparticles. Firstly, the collected macrophage membrane vesicles were mixed with nanoparticle cores with a membrane protein-to-polymer weight ratio of 1:1, and the mixture was sonicated with a bath sonicator at a frequency of 42 kHz and a power of 100 W for 2 min. Then, the mixture was extruded 15–20 times through a mini-extruder to obtain MM@Lips and MM@Lips-SHP1i nanoparticles. Finally, the resulting solution was centrifuged at 10,000 × g for 30 min to remove the uncoated membrane. The prepared macrophage membrane biomimetic nanoparticles are stored at 4 °C for further use.

The size/polydispersity coefficient (PDI) and zeta potential of MM vesicles, Lips, Lips-SHP1i, MM@Lips and MM@Lips-SHP1i were measured by dynamic light scattering detector (DLS) (Nano ZS90; Malvern, UK). All measurements were done in triplicate at room temperature. The morphology of MM vesicles, Lips and MM@Lips-SHP1i were visually observed using a transmission electron microscope (TEM) (JEM-2100F; JEOL, Japan). To verify the successful synthesis of Lips-SHP1i, the ultraviolet–visible absorption spectrum of SHP1i, Lips, Lips-SHP1i, MM vesicles, MM@Lips and MM@Lips-SHP1i were measured by UV–vis spectrophotometer. The particle sizes of MM@Lips-SHP1i nanoparticles dispersed in PBS and 10% mouse serum were determined over a span of 7 days, to evaluate the stability of biomimetic macrophage membrane nanoparticles.

### Membrane protein characterization

The surface membrane proteins were characterized by polyacrylamide gel electrophoresis (SDS-PAGE). Briefly, the Lips-SHP1i, macrophages, macrophage membrane vesicles and MM@Lips-SHP1i were prepared in a RIPA buffer supplemented with protease inhibitor and quantified by the BCA Protein Assay (Beyotime; China). Then, the samples were mixed with 5 × loading buffer before heating at 100 °C for 5 min. Approximately, 30 μg proteins for each sample were loaded into each well in 12% SDS-PAGE and run at 100 V for 2 h. After that, the SDS-PAGE gel was stained by Coomassie Blue Staining Kit (Beyotime; China) according to the provided protocol until imaged with investigator prolmage.

The specific surface markers on macrophage, macrophage membrane and MM@Lips-SHP1i were determined by Western blotting. Specifically, the proteins were transferred from the gel to the poly (vinylidene diflfluoride) membranes followed by blocking for 2 h with 5% skimmed milk powder in tris-buffered saline after the electrophoresis. Then, membranes were incubated overnight at 4 °C with primary antibodies, including CD36, SRA, TLR4, IL-6R, CD130, TNFR1, IFNGR1. Followed by incubation of a secondary HRP-conjugated affinipure goat anti-rabbit IgG (H + L) antibody (Proteintech; China) at room temperature for 1 h, specific bands were visualized using an enhanced chemiluminescent detection kit (NCM Biotech, China) under a chemiluminescence/fluorescence image analysis system (Tanon 5200, China).

The macrophage phenotype was also confirmed by evaluating the expression of CD80 (M1 macrophage phenotype marker) and CD206 (M2 macrophage phenotype marker) by Confocal Laser Scanning Microscope (CLSM). In brief, RAW264.7 cells were seeded in a 6-well plates containing cell slide at a density of 1 × 10^4^ cells per well. After overnight incubation, M1/M2 macrophages were polarized in vitro. After polarization, cells were fixed with 4% paraformaldehyde and subsequently incubated with 0.3% TritonX-100 for 30 min. After washing with PBS, the cells were blocked with blocking solution (10% NGS, 0.3% TritonX-100) for 2 h at room temperature. Then, cells were incubated overnight at 4 °C with primary antibodies CD80 or CD206. Followed by incubation of a fluorescence secondary antibody for 2 h, the nuclei were counterstained with DAPI staining solution. Finally, the slides were sealed with anti-fluorescence quenching agent, and the cells were observed and photographed under CLSM.

### oxLDL and LPS binding studies

To quantify oxLDL clearance rate by MM@Lips, MM@Lips (0.5 mg) were mixed with oxLDL of varying amount (10, 20, 30, 60 μg), respectively, in 1 × PBS containing 10% FBS [19]. In a parallel experiment, the removal amount was studied by fixing oxLDL amount of 60 μg but varying the amount of MM@Lips at 0.1, 0.2, 0.3, 0.4 and 0.5 mg, respectively. In both cases, the mixtures were incubated for 30 min and then centrifuged at 20,000 × g for 15 min to pellet the nanoparticles. The free oxLDL content in the supernatant was measured by using enzyme linked immunosorbent assay (ELISA) kit according to manufacturer’s instructions. All experiments were performed in triplicate.

Similarly, to quantify LPS clearance rate by MM@Lips, MM@Lips (0.5 mg) were mixed with different amout of LPS (5, 10, 25, and 50 ng), respectively. In parallel, 50 ng of LPS was mixed with different concentrations of MM@Lips (0.1, 0.2, 0.3, 0.4 and 0.5 mg). After incubated for 30 min, the free LPS content in the supernatant was quantified using limulus amebocyte lysate (LAL) assay according to the manufacturer’s instructions. All experiments were performed in triplicate.

### Cell culture

The RAW 264.7 macrophage cells and Human Umbilical Vein Endothelial Cells (HUVECs) were purchased from Cell Bank/Stem Cell Bank, Chinese Academy of Sciences. The cells were cultured in Dulbecco’s modified Eagle’s medium (DMEM) supplemented with 10% fetal bovine serum (Gibco, USA), penicillin (100 U/mL) and streptomycin (0.1 mg/mL). Cells were kept in an incubation chamber at 37 °C and 5% CO_2_ with a humidified atmosphere.

### Cytotoxicity evaluation

In vitro cytotoxicity of Lips, Lips-SHP1i, MM@Lips, MM@Lips-SHP1i were evaluated with RAW264.7 and HUVECs cells by MTT assays, respectively. Mainly, the cells were seeded in 96-well plates (1 × 10^4^ cells per well). After incubation for 24 h, fresh DMEM medium containing varying concentrations of Lips, Lips-SHP1i, MM@Lips, MM@Lips-SHP1i (0.1, 0.2, 0.3, 0.4, 0.5, 0.6 mg/mL) were incubated with cells for another 24 h. After discarding the medium, 100 μL of 1 mg/mL MTT solution was added into each well and incubated for 4 h in dark at 37 °C. Afterward, 100 μL of dimethylsulfoxide (DMSO) was introduced to dissolve the formed formazan crystals in each well. The absorbance at 490 nm was recorded by microplate reader (Thermo Scientific, USA). Each experiment was conducted 3 times in parallel. The cell viability was calculated by GraphPad Prism 9 according to their absorbances at 490 nm in each well.

### MM@Lips-SHP1i NPs escape clearance from immune system

To verify the effect of macrophage membrane coating in evading clearance from the immune system, fluorescently labeled nanoparticles were incubated with RAW264.7 macrophages to observe the phagocytosis reduction by macrophages. To prepare the fluorescently labeled nanoparticles, 1 wt % Coumarin-6 (excitation/emission wavelength = 466/504 nm) was loaded into blank liposomes. Briefly, RAW264.7 cells were seeded in 6-well plates at a density of 1 × 10^4^ cells per well and cultured overnight. Then, the media were replaced with fresh media containing 100 μg Lips-C6 NPs and MM@Lips-C6 NPs. After incubation for different times (0.25, 0.5, 1, and 2 h), the cells were washed with PBS for 3 times and fixed by paraformaldehyde for 15 min at room temperature. Subsequently, the cells were washed with PBS for three more times and the nuclei were stained with DAPI. After washing with PBS for 3 times again, the cells were observed via inverted fluorescence microscope.

### Inhibition of macrophage foaming *in vitro*

RAW264.7 macrophages were seeded in a 24-well plate (1 × 10^4^ cells per well). After incubation for 12 h, the cells were co-treated with oxLDL (60 μg/mL) and Lips, Lips-SHP1i, MM@Lips, MM@Lips-SHP1i NPs for 24 h, respectively. The degree of macrophage foaming was evaluated by oil red O (ORO) staining and the determination of intracellular total cholesterol (TC) concentration.

The ORO staining in macrophages was carried out according to the instructions. Specifically, the saturated oil red O stock solution was added to distilled water in a ratio of 3:2 (oil red O: distilled water) and filtered twice with filter paper before use. After fixed with 4% paraformaldehyde at room temperature for 15 min and washed with PBS, the macrophages were stained by freshly prepared ORO working solution for 15 min, and rinsed with 60% isopropanol. After washing with PBS, the cells were observed under microscope. The obtained images were further analyzed with Image J software.

### Inhibition of proinflammatory cytokines, ROS and iNO by LPS neutralization *in vitro*

RAW 264.7 macrophages were seeded in a 24-well plate at a density of 1 × 10^5^ cells/well and cultured overnight. Then the cells were co-treated with LPS (20 ng/mL) and Lips, Lips-SHP1i, MM@Lips, MM@Lips-SHP1i NPs for another 24 h. After incubation, the concentrations of proinflammatory cytokines including TNF-α, IL-6, IFN-γ in the supernatant were quantified using ELISA kits.

Furthermore, the ROS levels were analyzed to confirm the ROS production. Briefly, RAW264.7 macrophages were cultured in 6-well plates at a density of 1 × 10^4^ cells/well for 12 h. After co-treatment of cells by LPS (20 ng/mL) and different groups (Lips, SHP1i, Lips-SHP1i, MM@Lips and MM@Lips-SHP1i NPs) for 4 h, cells were rinsed and treated with 10 µM 2′,7′-dichlorofluorescin diacetate (DCFH-DA) in PBS for 30 min. Afterwards, the cells were washed with PBS for 3 times and the intracellular ROS generation was observed by Inverted Fluorescence Microscope. Results were analyzed using Image J software (version 7.6.1).

Production of intracellular nitric oxide (iNO) was also used to evaluate the influence of LPS neutralization with MM@Lips-SHP1i. Briefly, 2 × 10^4^ RAW264.7 macrophages were seeded in each well of a 96-well plate and incubated overnight. Then, 180 μL of medium containing 20 ng/mL of LPS was added to each well, followed with the addition of 20 μL of SHP1i, Lips-SHP1i, MM@Lips or MM@Lips-SHP1i, respectively. As control, 20 μL of PBS was added into control wells. Cells without any treatment served as the background. The plate was incubated at 37 °C for 24 h and the iNO productions were measured by a nitric oxide detection kit. The above-mentioned cell supernatant was collected, and 50 μL of the supernatant, 50 μL of Griess Reagent I and 50 μL of Griess Reagent II were added to each well, respectively. Finally, the absorbance value of each well at 540 nm was read with a microplate reader, and the concentration of nitric oxide in each sample was calculated according to the standard curve.

### Detection of scavenger receptor expression on macrophages

RAW264.7 macrophages were seeded in a 6-well plate at a density of 1 × 10^5^ cells/well and cultured overnight. After co-treated with oxLDL and SHP1i, Lips-SHP1i, MM@Lips, MM@Lips-SHP1i NPs for 24 h, respectively, the total cell protein of each group was extracted according to the previous steps. Western Blot was used to evaluate the expression of scavenger receptors (mainly CD36 and SRA) on the surface of macrophages in each group.

### Activated macrophage apoptosis assay

Briefly, RAW264.7 macrophages were cultured in 6-well plates at a density of 1 × 10^4^ cells/well for 12 h. After treated by oxLDL and SHP1i, Lips-SHP1i, MM@Lips or MM@Lips-SHP1i NPs for 24 h, the cells were collected by trypsinization without EDTA. Then the cells were washed twice with PBS and suspended in 500 μL of binding buffer. Subsequently, after adding 5 μL of Annexin V-fluorescein isothiocyanate (V-FITC) and 5 μL of propidium iodide (PI) and reacting for 15 min at room temperature, cells were analyzed by flow cytometer to determine the apoptosis rates. Results were analyzed using FlowJo software (version 7.6.1).

### *In vitro* efferocytosis assay

*In vitro* efferocytosis assays were performed by cell tracker. Briefly, RAW264.7 macrophages were labelled with CellTracker Red and pretreated with SHP1i, Lips-SHP1i, MM@Lips or MM@Lips-SHP1i for 30 min. To produce labelled apoptotic cells, RAW264.7 cells labelled with CellTracker Green were incubated with tumor necrosis factor-α (TNF-α) for 24 h. Then, apoptotic cells were plated in 6-well dishes at a density of 1.5 × 10^5^ cells per well and RAW264.7 cells were added at a density of 3 × 10^5^ cells per well. They were co-incubated for 2 h in serum-free media. Efferocytic activity was observed by fluorescence microscopy and evaluated by Image J software (version 7.6.1).

### Animals

Female C57BL/6 mice were obtained from the Animal Center of Xuzhou Medical University. Female apolipoprotein E knockout (ApoE-/-) mice aged 8 weeks were purchased from GemPharmatech Co., Ltd. (Nanjing, China). All animals were maintained under standard housing conditions and all animals were acclimatized for at least 3 days before the experiments started. All animal protocols were approved by the Ethics Committee of Xuzhou Medical University.

### Blood half-life determination

Six C57BL/6 mice aged 8 weeks, were randomly divided into two groups. In each group, 200 μL of Lips-C6 or MM@Lips-C6 nanoparticles (5 mg/mL) were injected intravenously. 10 μL of blood was quickly collected from the tail of the mouse at different time points post-injection (5 min, 15 min, 30 min, 1 h, 2 h, 4 h, 6 h, 8 h, 12 h, 24 h, and 48 h). The collected blood was immediately diluted with 10 μL of 2 mM EDTA-2 K PBS solution. The fluorescence intensities of the samples were measured with a fluorescence microplate reader. Fitting half-life curve and half-life (T_1/2_) was calculated according to the obtained fluorescence intensities with the time passing by.

### Construction of AS in ApoE-/- mice

Eight-week-old female ApoE-/- mice were fed a normal diet for 12 weeks in the negative control group while a high-fat diet (HFD) containing 20% fat and 1.25% cholesterol was given to another group to induce atherosclerosis.

### *In vivo* targeting of atherosclerotic plaque

Eight-week-old female ApoE-/- mice fed with high fat diet for 3 months were administered with Lips-C6 and MM@Lips-C6 via the tail vein, respectively. After 2 h, the mice were euthanized and subsequently perfused with PBS to remove the blood and unbound dyes. The aortas were isolated for imaging and fluorescence quantitative analysis using an chemiluminescence/fluorescence image analysis system.

### *In vivo* treatment of AS in ApoE-/- mice

ApoE-/- mice were randomized into 6 groups (7 mice per group) and injected with saline, SHP1i, Lips-SHP1i, MM@Lips or MM@Lips-SHP1i NPs every 4 days for 4 weeks. Mice in negative control group were given normal diet and those in other groups were fed with high fat diet. At the end of treatment, the ApoE-/- mice were euthanized and the degree of pathological evolution were evaluated by measuring the lesion area of the aorta from the heart to the iliac bifurcation. To determine the extent of atherosclerosis at the aortic root, aortic arch, and brachiocephalic artery, Oil Red O (ORO) staining was performed to confirm the formation of atherosclerotic plaque in mice.

Furthermore, quantitative analysis of atherosclerotic plaques was also determined by ORO staining with sequential 10 cryosections at 100 μm intervals from the aorta tissues. Subsequently, for histological analysis, aortic root, aortic arch and brachiocephalic artery from mice with various treatments were collected for hematoxylin–eosin (H&E) staining, Masson’s trichrome staining and toluidine blue staining. For immunohistochemistry analysis, sections of aortic root, aortic arch and brachiocephalic artery were incubated with antibodies, including CD68, CD14, MMP-9, and α-SMA, CD31 and KI67, respectively.

### *In vivo* efferocytosis assay

To confirm the interruption of MM@Lips-SHP1i NPs to CD47-SIRPα signaling in vivo, sections were co-stained with phospho-SHP1 and Mac-3, respectively, to assess the lesional SHP-1 activity. The phospho-SHP1 area was quantified and normalized to the Mac-3 area. To assess apoptotic cells in lesions, sections were stained for cleaved caspase-3. The percentage of cleaved caspase-3 + area was calculated and divided by the total atherosclerotic plaque area. To study the efferocytosis of apoptotic cells by macrophages, sections were co-stained with cleaved caspase-3 and Mac-3. And then the *in vivo* phagocytic index was calculated by manually counting the number of free apoptotic cells versus phagocytosed (macrophage-associated) apoptotic cells.

### Safety evaluation

During the above treatment, the changes of body weight of mice in all groups were recorded. At the endpoint of experiment, all mice were sacrificed, and the heart, livers, spleen, lungs, and kidneys were processed for histological study. In addition, the blood was collected to quantitate the immune-associated cells, such as red blood cells (RBCs), white blood cells (WBCs), platelets (PLTs), and hemoglobin (HGB). The serum was also collected for blood chemistry analysis, including the liver function biomarkers (ALT and AST), kidney function biomarkers (UREA and CREA).

In addition, the biocompatibility of MM@Lips-SHP1i was also evaluated by hemolysis assay [22]. Briefly, fresh mouse blood samples were collected and centrifuged to separate RBCs. After washed several times and diluted with saline, 200 μL of RBCs suspension was mixed with 800 μL of different nanoparticles. Red blood cells treated with the same volume of deionized water and saline were set as positive and negative controls. After incubation at 37 °C for 2 h, the mixture was centrifuged at 5000 rpm for 5 min, and the absorbance of the supernatant at 541 nm was measured by UV–vis spectrophotometer. The hemolysis rate of each sample was calculated by the following formula:

$$\mathrm{Hemolysis}(\mathrm{\%})=\frac{{\mathrm{A}}_{\mathrm{sample}}-{\mathrm{A}}_{\mathrm{negative\,control}}}{{\mathrm{A}}_{\mathrm{positive\,control}}-{\mathrm{A}}_{\mathrm{negative\,control}}}\times 100\mathrm{\%}$$

### Statistical analysis

The data obtained by at least three independent repeated experiments were presented as the mean ± standard deviation in this study. SPSS 22.0 software was used for the statistical analysis. One-way analysis of variance (ANOVA) was utilized for revealing differences among the groups, and when the overall difference was statistically significant, pairwise comparison was performed with least significant difference (LSD) method inspection. Values of *P < 0.05, **P < 0.01 and ***P < 0.001 were applied to annotate statistical significance.

## Results and discussion

### Preparation and characterization of MM@Lips-SHP1i

**Scheme 1.**
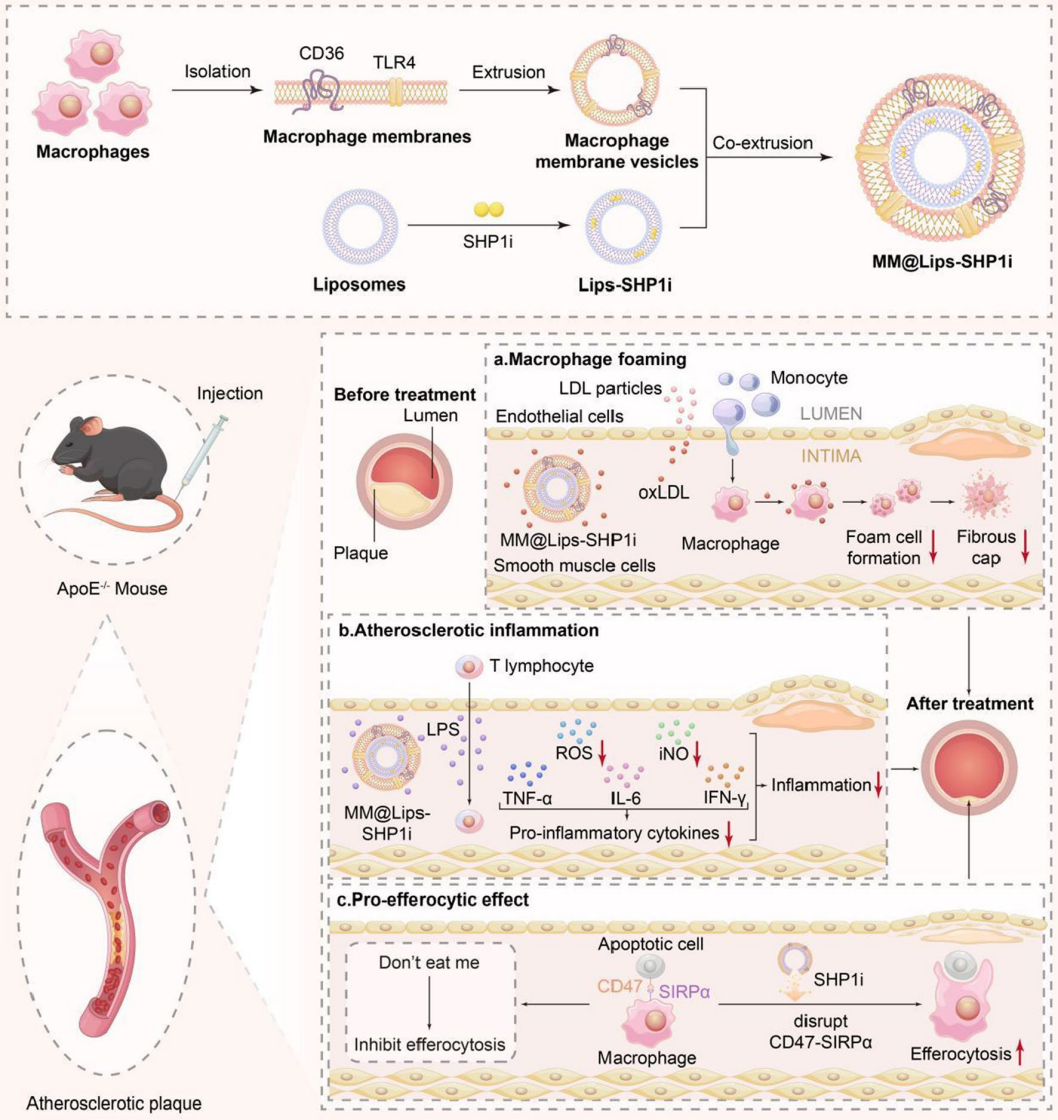
Schematic illustration of preparation of MM@Lips-SHP1i NPs and synergistic treatment for atherosclerosis

**Fig. 1:**
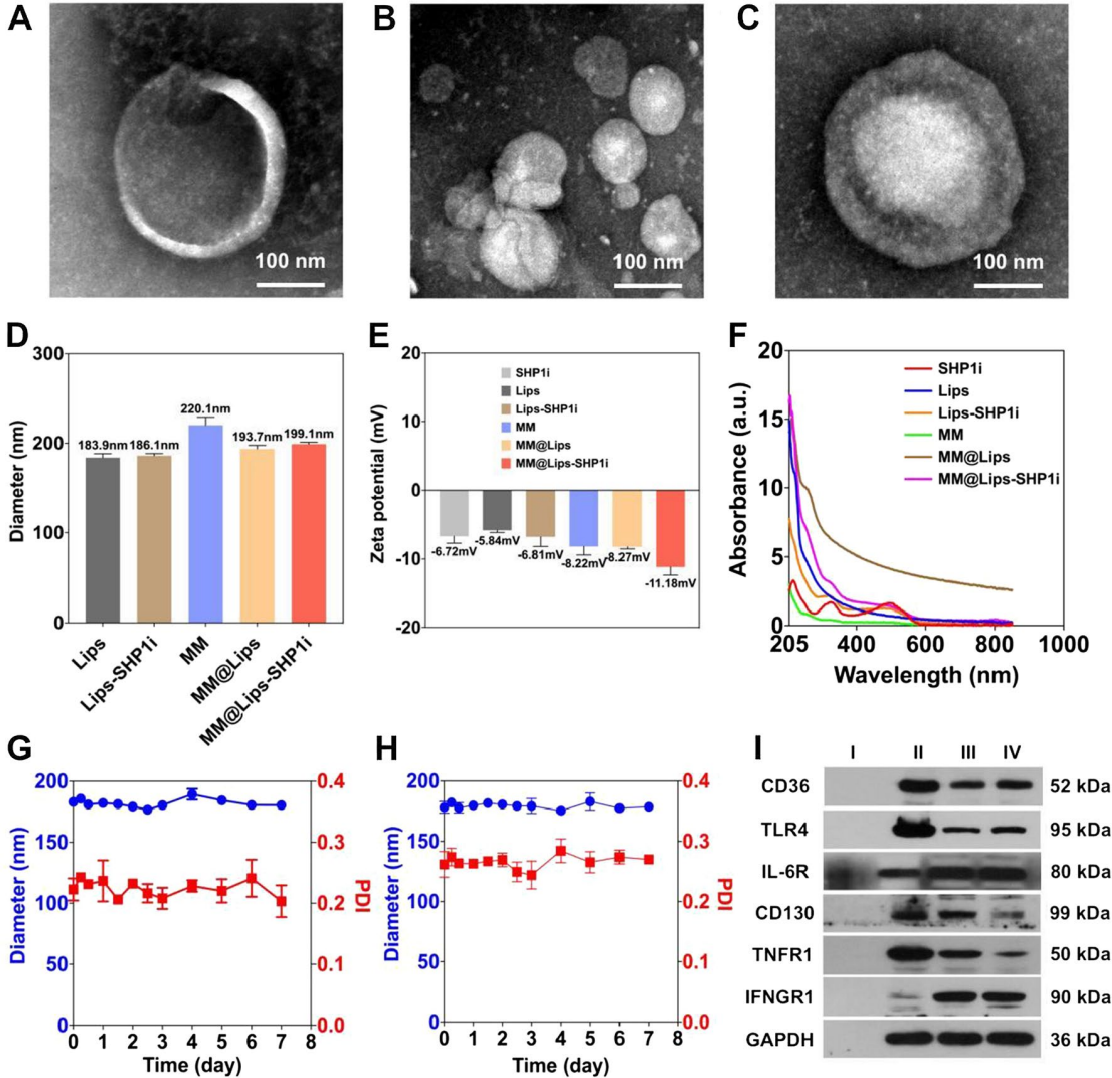
Characterizations of MM@Lips-SHP1i NPs: **A** TEM image of macrophage membrane vesicles; **B** TEM image of blank liposomes; **C** TEM image of MM@Lips-SHP1i; **D** Hydrodynamic diameters of Lips, Lips-SHP1i, MM, MM@Lips and MM@Lips-SHP1i; **E** Zeta potential of SHP1i, Lips, Lips-SHP1i, MM, MM@Lips and MM@Lips-SHP1i; **F** UV–vis absorption spectra of SHP1i, Lips, Lips-SHP1i, MM, MM@Lips and MM@Lips-SHP1i; **G** The size stability of MM@Lips-SHP1i NPs over a span of 7 days in PBS and **H** in 10% mouse serum. **I** Representative AS-related protein bands of Lips-SHP1i (I), macrophages (II), macrophage membranes (III) and MM@Lips-SHP1i (IV) using western blotting

For the preparation of MM@Lips-SHP1i nanoparticles, macrophage membrane was first extracted from RAW264.7 macrophage cells by hypotonic lysis, mechanical destruction, and differential centrifugation. To verify the successful isolation, the cell membrane and the nucleus were labeled with Dil and DAPI, respectively. As shown in Additional file 1: Figure S1, the red fluorescence emissions from cell membrane and the blue fluorescence emissions from nucleus were completely overlapped in macrophages before extraction. A series of extraction steps later, only the red fluorescence from the membrane could be observed, indicating the macrophage membrane was successfully separated. After the liposome loading with SHP1i was prepared by thin film dispersion method, macrophage membrane biomimetic nanoparticles were obtained by mechanical co-extrusion method as shown in Scheme 1. The extracted macrophage membranes showed distinct vesicle structure (Fig. 1A), and the blank liposomes showed uniform spherical structure (Fig. 1B). After successful coating of cell membrane onto liposome-SHP1i, a uniform core–shell spherical nanostructure was displayed, and each nanocore was surrounded by a monolayer membrane with a thickness of about 7 nm, which is consistent with the thickness of the membrane (Fig. 1C).

To further confirm such membrane coating, the hydrodynamic diameters (Dh) and Zeta potentials were determined. The average hydrodynamic diameter of Lips-SHP1i was increased from 186.1 ± 2.5 nm to 199.1 ± 2.0 nm after macrophage membrane coating (MM@Lips-SHPli) (Fig. 1D). In terms of zeta potential, MM@Lips-SHP1i exhibited a surface charge of approximately of -11.18 mV, which was more negative than Lips-SHP1i (− 6.81 mV) and macrophage membrane vesicles (− 8.22 mV), indicating the proper membrane coating (Fig. 1E). UV–vis spectroscopy validated SHP1i with characteristic absorption peaks at 230 nm, 320 nm and 490 nm, and lips-SHP1i and MM@Lips-SHP1i retained the above three characteristic absorption peaks, confirming the successful loading of SHP1i (Fig. 1F). The SHP1i loading efficiency (LE) and encapsulation efficiency (EE) in MM@Lips-SHP1i were determined to be 34.1% and 90%, respectively. Furthermore, a relatively constant hydrodynamic diameter of MM@Lips-SHP1i dispersed in PBS and in 10% mouse serum over a span of 7 days showed their good stability (Fig. 1G, H).

Next, we performed SDS-PAGE gel electrophoresis and Western Blot analysis to study the preservation of surface membrane proteins of macrophage membranes of MM@Lips-SHP1i, which is critical for their role in the progress inhibition of AS. SDS-PAGE gel electrophoresis result showed that the protein bands of macrophages, macrophage membranes and MM@Lips-SHP1i almost appeared in the same position (Additional file 1: Figure S2A). Western Blot analysis further confirmed the presence of key membrane antigens related to the progression of AS were retained in MM@Lips-SHP1i, including CD36 and SRA (oxLDL receptor), TLR4 (LPS receptor), IL-6R and CD130 (IL-6 receptor), TNFR1 (TNF-α receptor), and IFNGR1 (IFN-γ receptor) (Fig. 1H and Additional file 1: Figure S2B). This provides a basis for MM@Lips-SHP1i to inhibit the progression of AS, and again proves the successful synthesis of MM@Lips-SHP1i. Furthermore, no obvious CD80 and CD206 fluorescence emissions were observed in our used macrophages confirmed the macrophage phenotype in current culture environment (Additional file 1: Figure S3).

### oxLDL and LPS binding analysis and inhibition of oxLDL-induced macrophage foaming by biomimetic nanoparticles *in vitro*

**Fig. 2:**
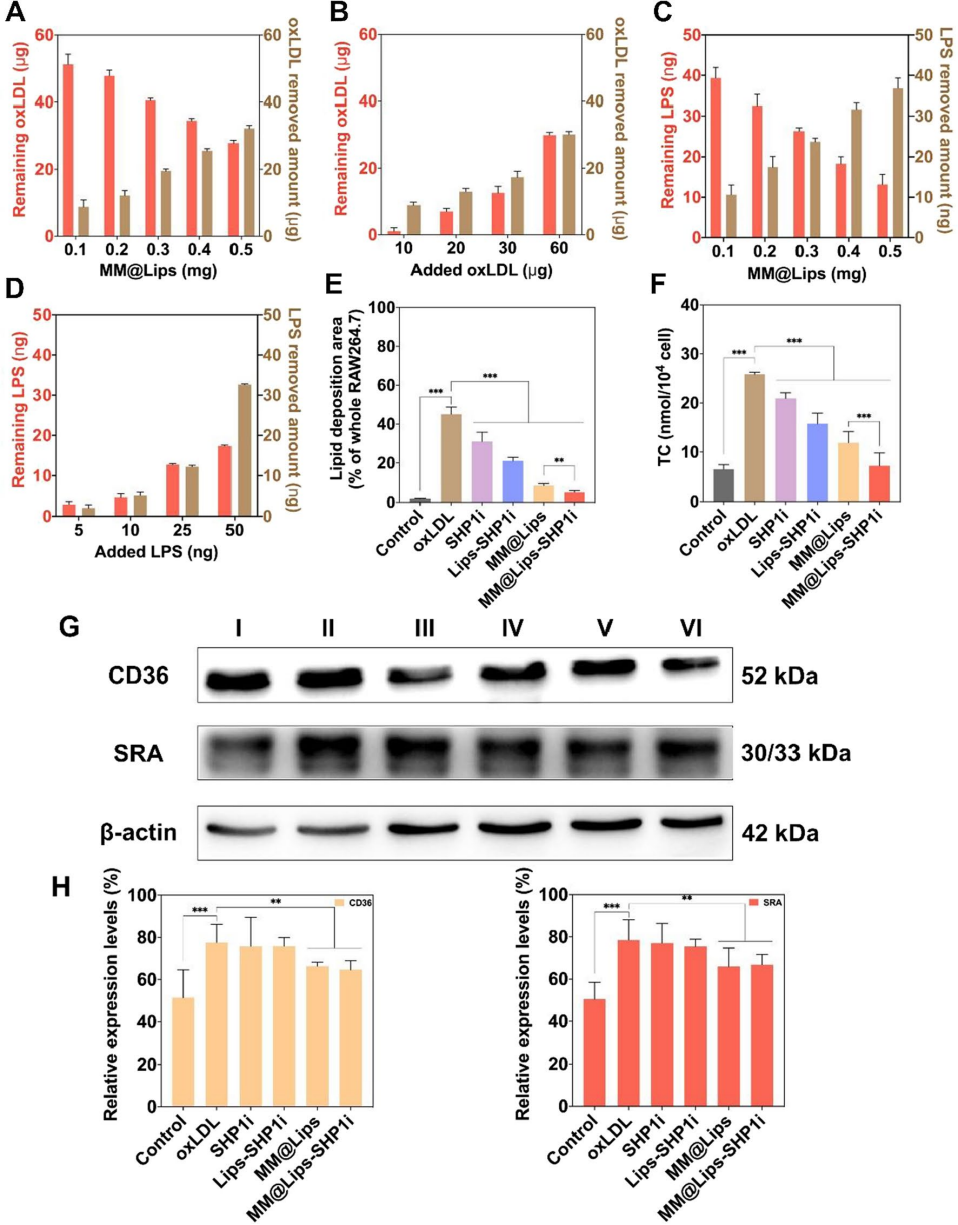
**A** Quantification of oxLDL removal with a fixed amount of oxLDL (60 μg) while varying the amount of added MM@Lips; **B** Quantification of oxLDL removal with a fixed amount of MM@Lips (0.5 mg) while varying the amount of added oxLDL; **C** Quantification of LPS removal with a fixed amount of LPS (50 ng) while varying the amount of added MM@Lips; **D** Quantification of LPS removal with a fixed amount of MM@Lips (0.5 mg) while varying the amount of added LPS; **E** The proportion of Oil Red O staining area in RAW264.7 cells after diferent treatments (**P < 0.01, ***P < 0.001); **F** Intracellular TC value of RAW264.7 cells after diferent treatments (***P < 0.001); **G** Western blotting was used to detect the scavenger receptor expression changes on RAW264.7 cells after different treatments (I: Control, II: oxLDL, III: SHP1i, IV: Lips-SHP1i, V: MM@Lips, VI: MM@Lips-SHP1i); **H** Quantification of CD36 and SRA relative expression levels (**P < 0.01, ***P < 0.001)

In early atherosclierotic plaques, when oxLDL uptake exceeds the metabolic capacity of macrophages, foam cell formation can be induced to promote the development of AS [23]. In order to verify the effect of biomimetic nanoparticles on macrophage foaming, their interaction with oxLDL was first studied. Considering the presence of binding receptors to oxLDL and LPS on macrophage membrane biomimetic nanoparticles, we evaluated their oxLDL and LPS removal capacity to effectively attenuate the interaction between macrophage cells and oxLDL or LPS. The oxLDL removal capacity of MM@Lips was quantified by two parallel experiments. As shown in Fig. 2A, B, with the increase of MM@Lips amount (0.1, 0.2, 0.3, 0.4, 0.5 mg) when fixing the concentration of oxLDL (60 µg), or with the increase of oxLDL amount (10, 20, 30, 60 µg) when fixing the concentration of MM@Lips (0.5 mg), an increased removal amount of oxLDL was observed and a removal capability of 62.3 µg/mg was obtained in the presence of 0.5 mg MM@Lips and 60 µg oxLDL. For LPS clearance, similar phenomenon was displayed and a removal capability of 69.46 ng/mg was obtained in the presence of 0.5 mg MM@Lips and 50 ng LPS (Fig. 2C, D), which was similar with MΦ-NPs reported by Thamphiwatana S. et al. [19].

Motivated by the above removal capacity of MM@Lips, we further studied their influence on the macrophage foaming in vitro. As shown in Additional file 1: Figure S4 and Fig. 2E, macrophages pretreated with oxLDL presented a large number of oil-red stained red fat droplets, while in the presence of SHP1i, MM@Lips, or MM@Lips-SHP1i, the proportion of oil red staining area was significantly reduced and the effect could be reached optimum in MM@Lips-SHP1i group. The same phenomenon was observed by measuring the TC values in macrophages after different treatments, illustrating the inhibition effect of MM@Lips-SHP1i on the formation of oxLDL-induced foam cells (Fig. 2F). To deeply confirm the role of macrophage membrane in such inhibition, western blot analysis was introduced to compare the expression changes of CD36 and SRA of RAW264.7 cells before and after different treatments. CD36, a membrane glycoprotein, is present in various types of cells, including monocytes, macrophages, microvascular endothelial cells, adipocytes, and platelets [24]. Macrophages interact with oxLDL via CD36, leading to the formation of foam cells, and triggering a signaling cascade of inflammatory responses, which are the initial critical phase of AS [24]. In addition, the early pathological changes of AS are also manifested by the overexpression of scavenger receptor A (SRA) on the surface of activated macrophages [25]. The binding between oxLDL and biomimetic nanoparticles might weaken the interaction between oxLDL and macrophages, down-regulating the expressions of CD36 and SRA on macrophages. As expected, the expressions of CD36 and SRA on the surface of oxLDL-treated RAW264.7 cells were up-regulated, which were significantly reduced after MM@Lips or MM@Lips-SHP1i treatment (Fig. 2G, H). It should be mentioned that SHP1i and Lips-SHP1i displayed no significant effect on such expression change, indicating the role of macrophage cell membrane of MM@Lips-SHP1i on the inhibition of the expressions of CD36 and SRA induced by oxLDL.

### Inhibition on the production of pro-inflammatory cytokines, ROS and iNO in LPS treated macrophages by MM@Lips-SHP1i

**Fig. 3:**
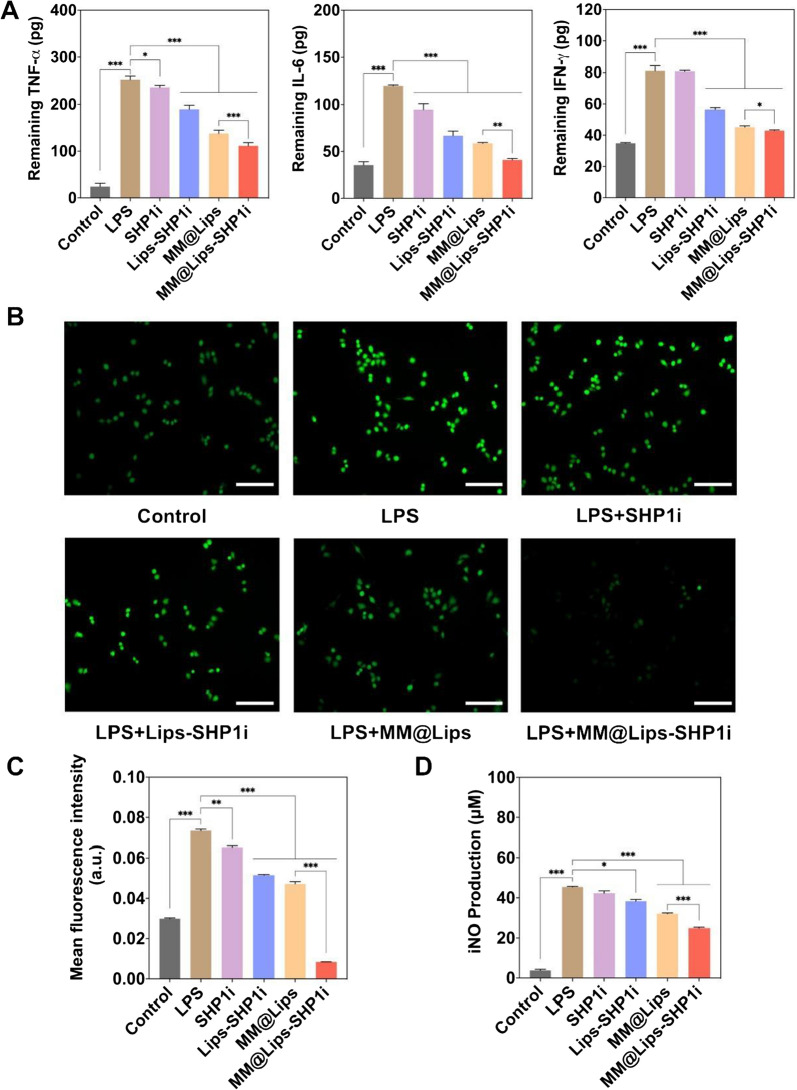
**A** The levels of pro-inflammatory cytokines, including TNF-α, IL-6 and IFN-γ in RAW264.7 supernatant after different treatments (*P < 0.05, **P < 0.01, ***P < 0.001); **B** Fluorescence images observed the effect of different nanoparticles on ROS generation in RAW264.7 cells. The cells were stained with DCFH-DA fluorescent probe and photographed under the fluorescence microscope (scale bar: 50 μm); **C** Analysis for mean DCFH-DA fluorescence intensity (**P < 0.01, ***P < 0.001); **D** Intracellular NO production after different treatments (*P < 0.05, ***P < 0.001)

Studies have shown that macrophages can specifically recognize and bind LPS through Toll-like receptor 4 (TLR4) to promote the production of pro-inflammatory cytokines, thereby accelerating the process of AS [26] [27]. Thus, the removal ability of biomimetic nanoparticles to LPS might favor the inhibition of the production of pro-inflammatory cytokines. To verify the effect of MM@Lips-SHP1i on macrophage inflammation, LPS pre-treated macrophages were used as inflammatory cell model in vitro. As shown in Fig. 3A, Lips-SHP1i, MM@Lips and MM@Lips-SHP1i all decreased the production of TNF-α, IL-6 and IFN-γ on LPS induced macrophages with a maximum inhibition effect in MM@Lips-SHP1i group.

In addition, it was reported that LPS could promote the production of intracellular ROS by promoting P-selectin expression [28] and increase iNO production by increasing the expression of inducible nitric oxide synthase (iNOS) [29]. The accumulation of ROS in atherosclerotic endothelial injury promotes macrophage foaming, stimulates their production of pro-inflammatory cytokines and chemokines, and enhances the inflammatory activity of macrophages. Excessive ROS accumulation also induces vascular cell damage, inflammatory cell recruitment, lipid peroxidation, metalloproteinase activation, and extracellular matrix deposition, which together lead to vascular remodeling [30] [31]. Nitric oxide (NO) is a multifunctional signaling molecule, which has a number of intracellular effects that lead to vasorelaxation, endothelial regeneration, inhibition of leukocyte chemotaxis, and platelet adhesion [32, 33]. Excessive production of NO can lead to endothelial cell dysfunction, leading to dysregulation of vascular tone, oxidative stress, and can also promote oxLDL uptake and enhance the release of pro-inflammatory cytokines from macrophages, promoting the development of atherosclerosis [34].

Thus, the ROS levels and NO production in macrophages with different treatment were detected. DCFH-DA fluorescent probe was applied to monitor the intracellular ROS generation. Compared with LPS group, the green fluorescence intensities were significantly reduced with the introduction of Lips-SHP1i, MM@Lips, and MM@Lips-SHP1i (Fig. 3B, C). ROS level of MM@Lips-SHP1i treated LPS induced macrophages was even lower than that of macrophages without any intervention, showing the excellent behavior of biomimetic nanoparticles on the inhibition of ROS generation. A similar result of iNO production was obtained (Fig. 3D), indicating that MM@Lips-SHP1i could reduce the production of iNO in macrophages by the clearance of LPS. Furthermore, liposomes alone showed no significant influence on the production of pro-inflammatory cytokines and ROS (Additional file 1: Figure S5). Thus, the better inhibition effect of MM@Lips-SHP1i on the production of pro-inflammatory cytokines and ROS can be ascribed to the removal capability of macrophage membrane to oxLDL and LPS, the increases of efferocytosis of macrophages by the role of SHP1i and the higher SHP1i delivery efficiency to cells by liposome. Liposomes, as nano-drug carriers, have a cell-like structure. In the process of drug delivery, after liposomes are adsorbed to the surface of the cell, the lipid bilayer of liposomes diffuses with the lipoidal cell membrane which allows the direct release of loaded drug, such as SHP1i into the cytoplasm to realize drug delivery [35]. J. Leeper also testified the enhanced therapeutic effect of SHP1i with the help of nanocarrier, SWNT and they ascribed this phenomenon to the delivery of pro-efferocytic SHP1i specifically to macrophages by SWNTs [18].

### Cytotoxicity, inhibition of oxLDL-induced apoptosis of macrophages, and pro-efferocytic effect of MM@Lips-SHP1i *in vitro*

**Fig. 4:**
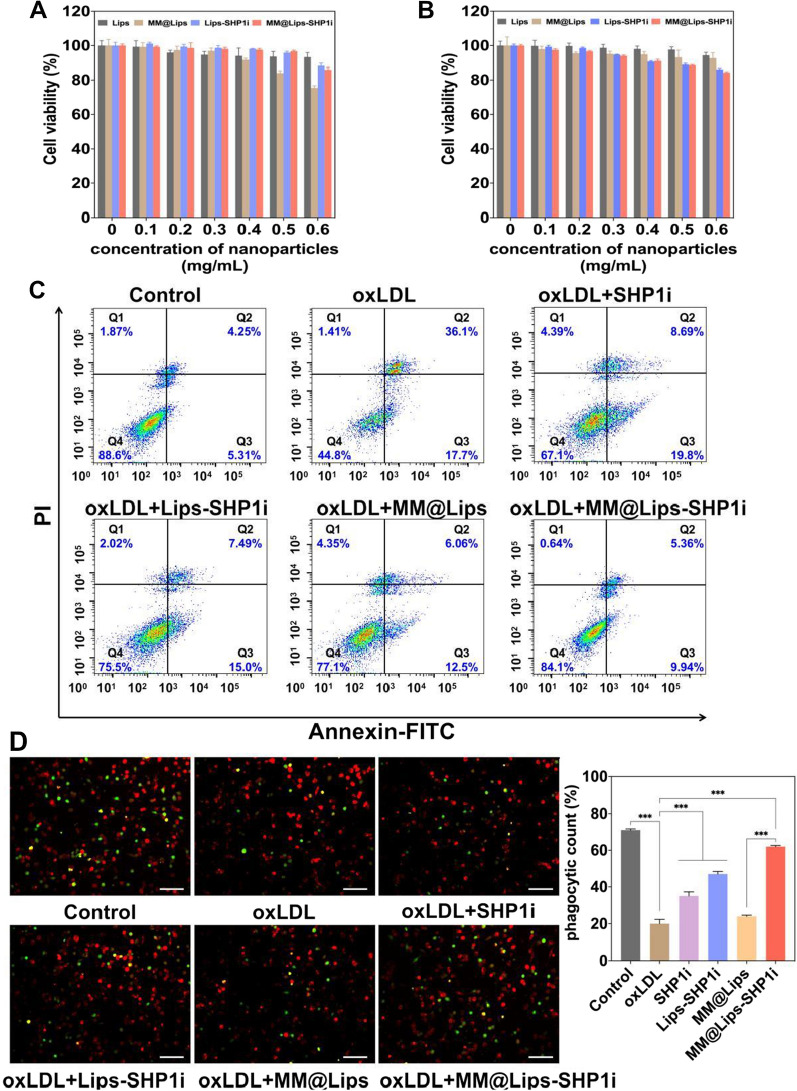
**A** Cell viabilities of RAW264.7 cells treated with different nanoparticles; **B** Cell viabilities of HUVECs treated with different nanoparticles; **C** Flow cytometric analysis of cell apoptosis after different treatments determined by the Annexin V-FITC/PI assay; **D** Fluorescence images observed pro-efferocytic effect in vitro after RAW264.7 macrophages were treated with different nanoparticles. The RAW264.7 macrophages were stained with CellTracker Red and apoptotic cells were stained with CellTracker Green, respectively (scale bar: 50 μm). The phagocytic index of RAW264.7 macrophages was calculated by Image J software (***P < 0.001)

MTT assay was used to evaluate the cytotoxicity of Lips, Lips-SHP1i, MM@Lips, MM@Lips-SHP1i to RAW264.7 and HUVECs. As shown in Fig. 4A, B, no significant cytotoxicities were observed in all groups under the concentration of 0.5 mg/mL. The presence of oxLDL could induce the apoptosis of macrophages which was confirmed by flow cytometry. The incubation of RAW264.7 with oxLDL for 24 h resulted in about 36.1% of cells underwent late apoptosis, and about 17.7% of cells underwent early apoptosis (Fig. 4C). However, SHP1i, Lips-SHP1i, MM@Lips and MM@Lips-SHP1i could decrease the proportions of both early and late apoptotic cells induced by oxLDL. The proportions of late apoptotic cells and early apoptotic cells in MM@Lips-SHP1i group were suppressed to 5.36% and 9.94%, respectively (Fig. 4C), showing that MM@Lips-SHP1i could effectively inhibit oxLDL-induced apoptosis of macrophages.

Studies have shown that when efferocytosis in atherosclerosis is enhanced, it can activate the clearance of apoptotic cells in the plaque, reduce the occurrence of secondary necrosis, and improve the stability of the plaque [36]. SHP1i as a SHP1 inhibitor can enhance the clearance of apoptotic cells by macrophages via blocking the CD47-SIRPα signaling pathway. Thus, macrophages labeled with CellTracker Red and oxLDL-induced apoptotic cells marked with CellTracker Green were imaged after different treatments in vitro. As shown in Fig. 4D, the phagocytic index of RAW264.7 macrophages without any treatment was about 71.98%, which was down to 20.43% with oxLDL treatment. But with the introduction of SHP1i, Lips-SHP1i, MM@Lips and MM@Lips-SHP1i, the phagocytosis indexes raised to 35.33%, 47.62%, 24.08% and 62.51%, respectively. Significant changes in all SHP1i containing nanoparticles except MM@Lips showed that such enhancement of efferocytosis effect of macrophages on apoptotic cells might come from the role of SHP1i, which was further strengthened with the macrophage membrane coating and liposome delivery.

### *In vivo* targeting of atherosclerotic plaques

**Fig. 5:**
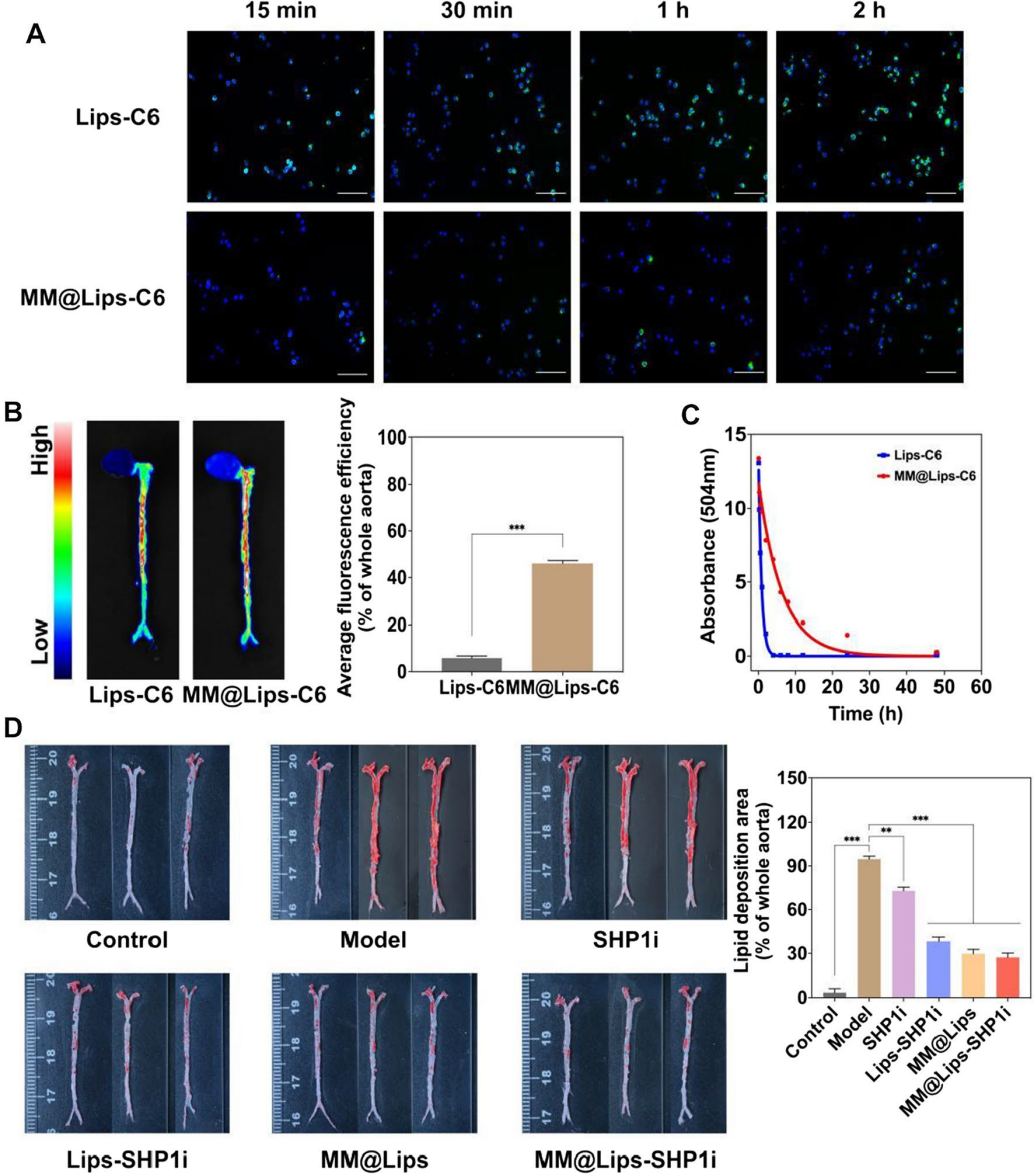
**A** Fluorescence images of the cellular uptake of Lips-C6 and MM@Lips-C6 by RAW264.7 macrophages to observe the immune escape ability (scale bar: 50 μm); **B** Aortic fluorescence images observed the targeting of Lips-C6 and MM@Lips-C6 to the atherosclerotic plaque (n = 3, ***P < 0.001); **C** The half-life of Lips-C6 and MM@Lips-C6 NPs; (D) ORO stained aorta tissues collected from atherosclerotic mice after different treatments (n = 3, **P < 0.01, ***P < 0.001)

As a chronic inflammatory disease, one of the key events in the development of AS is the activation of inflammatory endothelial cells, which express a series of adhesion molecules that can recruit macrophages to aggregate to atherosclerotic plaques [37]. Taking this into account, macrophage membrane biomimetic nanoparticles that inherit the biological characteristics of macrophages might possess the potential ability to actively target AS plaques. Thus, we firstly investigated the immune escape ability of macrophage membrane biomimetic nanoparticles. MM@Lips nanoparticles loading with coumarin-6 were incubated with macrophages and it can be seen that compared with Lips-C6, MM@Lips-C6 greatly reduced their uptake by macrophages (Fig. 5A), indicating the excellent immune escape ability of MM@Lips with the help of coated macrophage membrane by escaping the phagocytosis of macrophages. Then, Lips-C6 and MM@Lips-C6 nanoparticles were injected into ApoE-/- mice through the tail vein, respectively, and the fluorescence imaging was performed on the aorta of the mice. Compared with Lips-C6, MM@Lips-C6 exhibited stronger fluorescence intensity at aortic atherosclerotic plaques, showing a higher accumulation efficiency (Fig. 5B). This suggests that macrophage membrane coating strategy can significantly enhance the targeting and accumulation rate of nanoparticles to the atherosclerotic plaque area. Indeed, the immune evasion ability exhibited by macrophages is essentially a consequence of their surface membrane proteins [38]. Macrophages can identify MM@Lips-SHP1i NPs as “self-particles” due to the “self-marker” CD47 expressed on the macrophage membrane. As a transmembrane protein, CD47 plays an important role in mediating cell proliferation, migration, phagocytosis, apoptosis, immune homeostasis and other related responses, and is expressed on all cell membranes [39]. After the innate immune checkpoint CD47-SIRPα interaction, they send a "don't eat me" signal to macrophages, which can ultimately prevent phagocytosis by inhibiting macrophage contraction, thereby helping them to achieve immune escape. Besides, functional molecules such as CD45, CD11a and glycans on the macrophage membrane also help to prevent the internalization/uptake of biomimetic nanoparticles by phagocytes cells, which promotes the nanoparticles smoothly gathering to plaques [40]. The intrinsic biocompatibility of macrophage membranes allows the crossing of biological barriers and avoids elimination by the immune [40], which have also been testified in a series of macrophage membrane coating biomimetic nanoparticles [11, 41–43]. Such immune escape and targeting ability also extended their blood half-life in mice. The half-life of Lips-C6 was determined to be about 1.24 h, while that of MM@Lips-C6 was about 7.82 h (Fig. 5C), showing macrophage membrane coating strategy can significantly prolong the blood circulation time of the nanoparticles. Upon fulfilling its mission of inflammatory sites’ homing and reticuloendothelial system evasion, the macrophage-membrane coating can be shed via morphological changes driven by extracellular microenvironment stimuli via the proton sponge effect [44, 45]. The Lips-SHP1i was then endocytosis to activated macrophages in AS plaques and the following released SHP1i could produce an enhanced efferocytosis effect.

### Therapeutic efficacy in atherosclerotic mice

ApoE-/- mice were treated for one month with different drugs (saline, SHP1i, Lips-SHP1i, MM@Lips, and MM@Lips-SHP1i) to evaluate their therapeutic efficacy in atherosclerotic mice. The weight of the mice was continuously recorded during the whole treatment period, and the data showed little effect on the weight of the mice with different treatments (Figure S6). At the end of treatment, the aortas of mice were collected and stained with ORO to clearly observe the atherosclerotic lesions of the entire aorta. The ApoE-/- mice in all groups were fed with high-fat food except control group. As shown in Fig. 5D, compared with saline-treated control group, the aortic vessels of mice in saline-treated Model group displayed a large number of ORO-stained red plaques on the aortic vessels, indicating intraplaque lipid deposition. After treated with SHP1i, Lips-SHP1i, or MM@Lips, the aortic plaques of the mice were all gradually reduced, with the best inhibition effect in MM@Lips-SHP1i group. The results showed that the highest average area ratio of plaque to vessel lumen was displayed in Model group (about 94.71%), which was decreased to 72.88%, 38.28%, 29.66%, and 27.22% in SHP1i, Lips-SHP1i, MM@Lips and MM@Lips-SHP1i group, respectively.

**Fig. 6:**
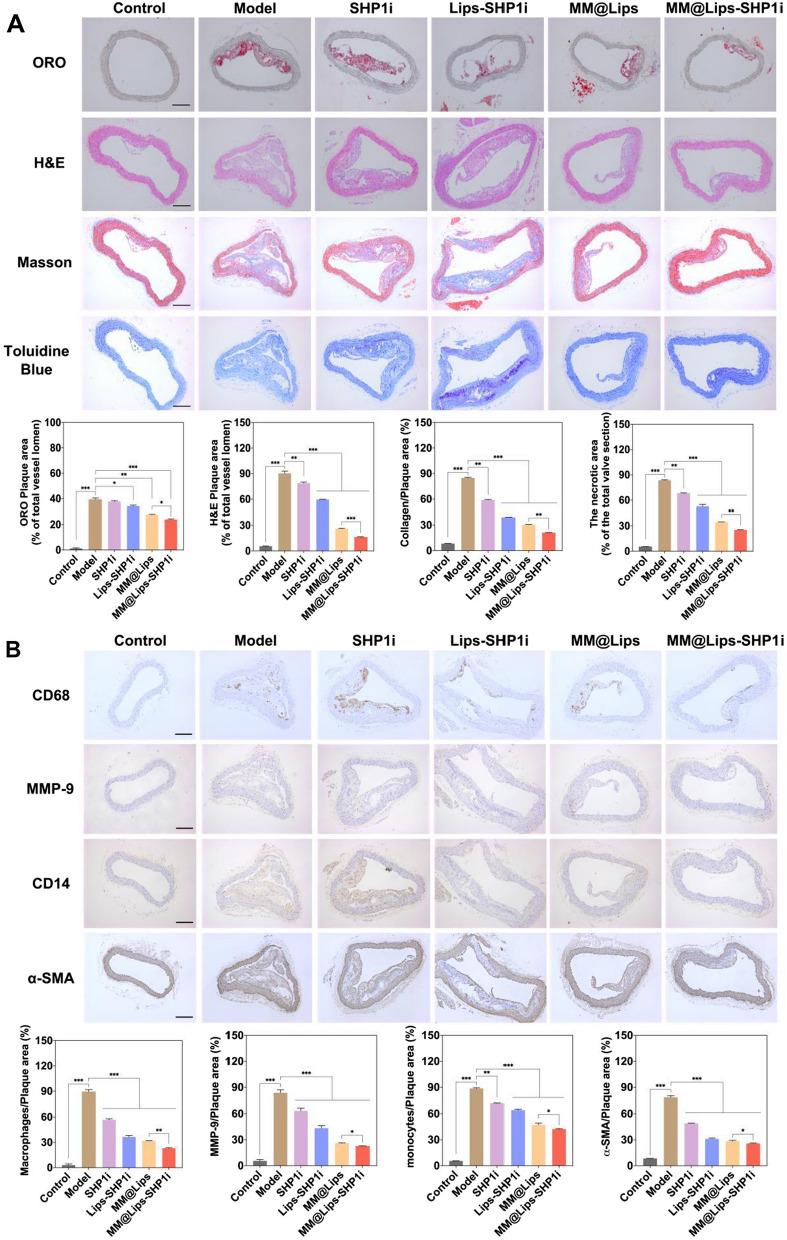
**A** Representative photographs and quantitative analysis of aorta root sections stained by ORO, H&E, Masson’s trichrome and Toluidine Blue (n = 3, scale bar: 200 μm, *P < 0.05, **P < 0.01, ***P < 0.001). **B** Representative photographs and quantitative analysis of aorta root sections stained by CD68 antibody, MMP-9 antibody, CD14 antibody and α-SMA antibody (n = 3, scale bar: 200 μm, *P < 0.05, **P < 0.01, ***P < 0.001)

Next, histological and immunohistochemical analysis was performed on the atherosclerotic plaques to further study the AS inhibition in all groups (Fig. 6A). ORO staining on the aortic roots of ApoE-/- mice was to observe the degree of plaque lesions in the vascular lumen. The results showed that the highest average area ratio of plaque to vessel lumen was displayed in Model group (about 39.52%), which was decreased to 37.69%, 34.20%, 27.69%, and 23.98% in SHP1i, Lips-SHP1i, MM@Lips and MM@Lips-SHP1i group, respectively. H&E staining of the aortic root revealed that the plaques in Model group were mostly necrotic nuclei. In contrast, the areas of plaques and necrotic nuclei were significantly reduced in MM@Lips-SHP1i-treated group. The accumulation of collagen in the plaque will increase the plaque area, resulting in further narrowing of the vascular lumen. Therefore, Masson staining was used to detect the change of collagen content in the plaque. It was shown that a large amount of collagen in the plaques of Model group, which was gradually decreased in SHP1i, Lips-SHP1i, MM@Lips and MM@Lips-SHP1i group. Toluidine blue staining can reflect the size of necrotic nuclei inside the plaque to assess the degree of plaque vulnerability. A large necrotic area with a large number of cholesterol crystals was shown in Model group and the necrotic area gradually decreased in SHP1i, Lips-SHP1i, MM@Lips, and MM@Lips-SHP1i group. All the above staining experiments indicated the excellent inhibition effect of MM@Lips-SHP1i on the AS progression.

Furthermore, we also analyzed the composition of atherosclerotic plaques by immunohistochemical staining (Fig. 6B). Analysis of CD68 (a marker of macrophages) showed that MM@Lips-SHP1i effectively reduced the number of macrophages in the plaque area, suggesting that MM@Lips-SHP1i effectively inhibited luminal narrowing due to obstruction of macrophage infiltration. Analysis of MMP-9 in the plaque area showed that the expression of MMP-9 in the plaque area was significantly reduced after MM@Lips-SHP1i treatment, suggesting that MM@Lips-SHP1i could stabilize atherosclerotic plaque. CD14 (a marker of monocyte) is also involved in the chronic inflammatory process of atherosclerosis, and analysis of CD14 showed that MM@Lips-SHP1i effectively reduced the number of monocytes in plaque areas. α-SMA analysis showed that the number of smooth muscle cells in plaque area decreased sharply after MM@Lips-SHP1i treatment, suggesting that MM@Lips-SHP1i can effectively inhibit the progression of atherosclerosis and the luminal stenosis caused by the growth of vascular smooth muscle cells. Similarly, analysis of CD31 (a marker of endothelial cell) confirmed that MM@Lips-SHP1i could significantly decrease the number of perivascular CD31 ( +) neovascularization, suggesting MM@Lips-SHP1i could not only maintain vascular endothelial integrity, but also display no toxicity to endothelial cells (Additional file 1: Figure S7). The results of KI67 (a marker of proliferative cell) study showed that MM@Lips-SHP1i effectively inhibited endothelial cell proliferation (Additional file 1: Figure S7).

**Fig. 7:**
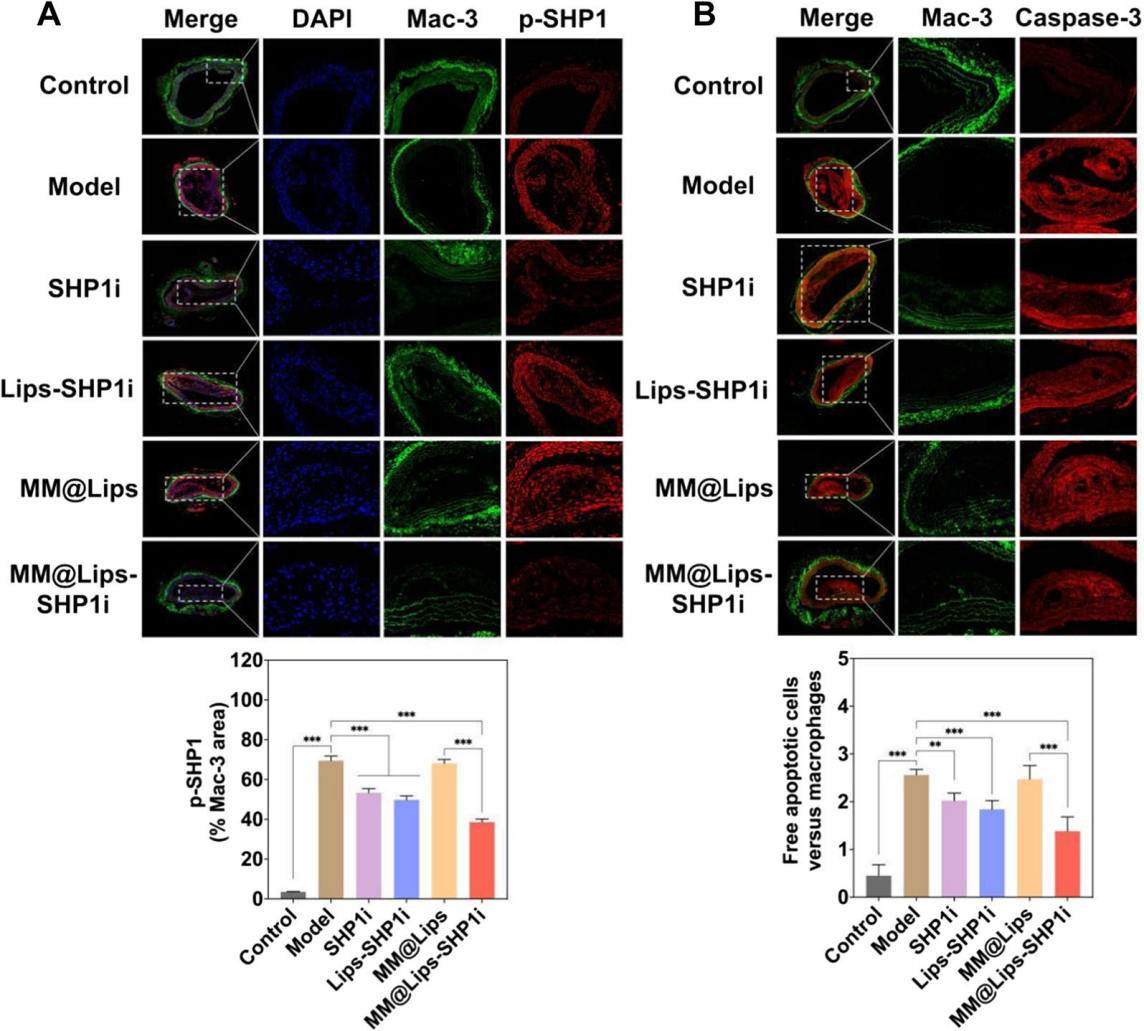
**A** Representative immunofluorescence images and quantitative analysis of aorta root sections co-stained by phosphorylated SHP-1 antibody and Mac-3 antibody to assess the lesional SHP-1 activity (scale bar: 200 μm, ***P < 0.001); **B** Representative immunofluorescence images and quantitative analysis of aorta root sections co-stained with cleaved caspase-3 and Mac-3 to study the efferocytosis of apoptotic cells by macrophages. The *in vivo* phagocytic index was determined by manually counting the number of free apoptotic cells versus macrophages (scale bar: 100 μm, **P < 0.01, ***P < 0.001)

Finally, we analyzed the activity of SHP-1 phosphorylation within plaques to verify the effect on macrophage efferocytosis *in vivo*. As shown in Fig. 7A, the activity of phosphorylated SHP-1 at the plaques in Model group was higher, and the activity of phosphorylated SHP-1 decreased gradually after treated with SHP1i, Lips-SHP1i and MM@Lips-SHP1i. The higher phosphorylated SHP-1 activity in MM@Lips group confirmed the above decrease in other groups might come from the effect of SHP1i. MM@Lips-SHP1i could disrupt the key effector of CD47-SIRPα downstream anti-phagocytic signaling in the atherosclerotic plaque area. Caspase-3 staining of the sections was employed to indicate the number of apoptotic cells in AS plaques and Caspase-3 and Mac-3 co-staining was used to study the efferocytosis of macrophages on apoptotic cells. The results showed that compared with other groups, the number of apoptotic cells (Additional file 1: Figure S8) and the ratio of free apoptotic cells to macrophages in AS plaques (Fig. 7B) were both lowest in MM@Lips-SHP1i group, indicating that the cellular efferocytosis of macrophages was significantly enhanced. The above experimental results all show that MM@Lips-SHP1i NPs has an excellent therapeutic effect on atherosclerosis via competing with macrophages in vivo to bind with oxLDL and LPS and enhancing the efferocytosis of macrophages. The detailed comparation between MM@Lips group and MM@Lips-SHP1i group would clearly show the synergistic effect through efferocytosis. Compared with MM@Lips, the MM@Lips-SHP1i has a better therapeutic effect in the inhibition of lipid deposition (*P* < 0.01, *P* = 0.005), intracellular total cholesterol (*P* < 0.001, *P* = 0.0009), the production of TNF-α (*P* < 0.001, *P* = 0.000056), IL-6 (*P* < 0.01, *P* = 0.009), IFN-γ (*P* < 0.05, *P* = 0.03), ROS (*P* < 0.001, *P* = 0.0007) and iNO (*P* < 0.001, *P* = 0.0001), and enhancing pro-efferocytic effect (*P* < 0.001, *P* = 0.0009) in vitro. Furthermore, compared with MM@Lips, MM@Lips-SHP1i also displayed a better treatment effect in vivo by histological and immunochemical analyses of the aorta, such as ORO plaque area (*P* < 0.05, *P* = 0.013), H&E plaque area (*P* < 0.001, *P* = 0.0006), collagen/plaque area ratio (*P* < 0.01, *P* = 0.003), the necrotic area (*P* < 0.01, *P* = 0.006), CD68 (*P* < 0.01, *P* = 0.007), MMP-9 (*P* < 0.05, *P* = 0.041), α-SMA (*P* < 0.05, *P* = 0.037), CD14 (*P* < 0.05, *P* = 0.02) and pro-efferocytic effect (*P* < 0.001, P = 0.00005).

### Biocompatibility of MM@Lips-SHP1i nanoparticles *in vivo*

**Fig. 8:**
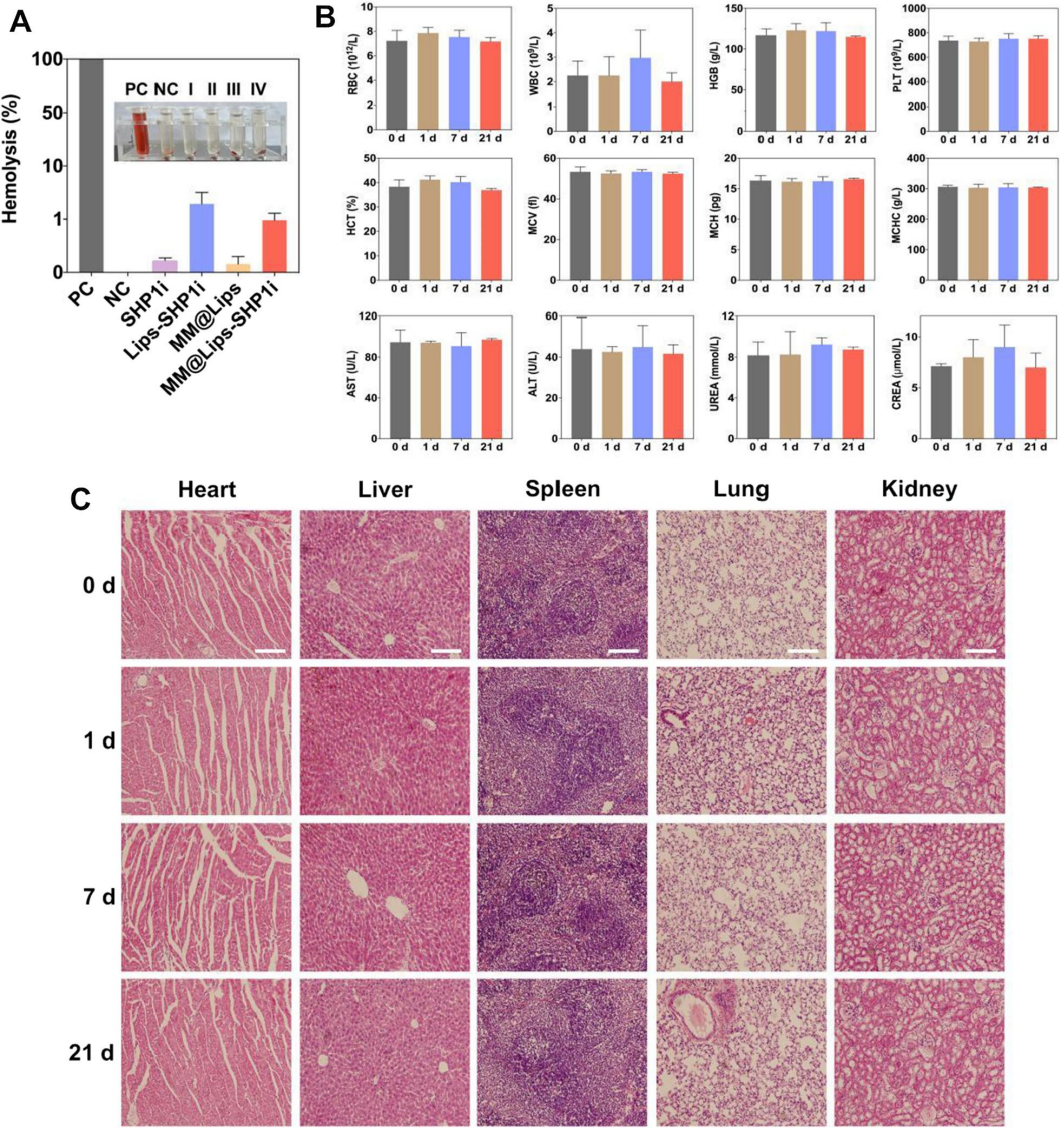
**A** The hemolysis of PC (positive control), NC (negative control), SHP1i (I), Lips-SHP1i (II), MM@Lips (III) and MM@Lips-SHP1i (IV) to mouse red blood cells (DI water as a positive control and saline as a negative control); **B** Mouse blood panel and serum biochemistry analysis before (0d, control) and after injection of MM@Lips-SHP1i for 1, 7, 21d; **C** H&E staining of collected organs including the heart, liver, spleen, lungs, kidneys of healthy mice after injected with MM@Lips-SHP1i at 1, 7 and 21 d and control mice (scale bar: 200 μm)

In order to systematically evaluate the biocompatibility of MM@Lips-SHP1i nanoparticles, we first evaluated them through hemolysis experiment. In the hemolysis experiment, when the nanomaterials were directly contacted with the red blood cells, the red blood cells would be destroyed to a certain extent, leading to the leakage of hemoglobin and hemolysis. The results in Fig. 8A showed that there was no obvious hemolysis in all samples, and the hemolysis rate of each group was lower than 5%.

Subsequently, blood routine and blood biochemical tests were performed on C57BL/6 mice after 1, 7 and 21 days of MM@Lips-SHP1i injection. Compared with control group, there were no significant differences in various indexes of mice after injected with MM@Lips-SHP1i (Fig. 8B). H&E staining analysis of the main organs (heart, liver, spleen, lung and kidney) of mice at different times post-injection of MM@Lips-SHP1i nanoparticles showed that the main organs of mice in each group kept normal tissue structure, without obvious organic lesions, inflammation or other abnormalities, indicating that MM@Lips-SHP1i had no obvious damage to the organs of mice (Fig. 8C).

## Conclusion

In this study, considering the key role of macrophages in AS, we developed macrophage membrane biomimetic nanoparticles MM@Lips-SHP1i to achieve the inhibition of AS progression by the inherent biological characteristics of macrophage membrane and the synergistic effect of SHP1i. The fabricated MM@Lips-SHP1i displayed good biocompatibility, long blood circulation time, immune escape ability and actively aggregation in atherosclerotic plaques. In AS plaque, MM@Lips-SHP1i competed with macrophages to bind oxLDL and LPS, reducing the foaming of macrophages and the release of proinflammatory cytokines. Meanwhile, the loaded anti-macrophage phagocytosis inhibitor SHP1i, blocked the CD47-SIRPα signaling pathway and promote the efferocytosis of macrophages on apoptotic cells. The above synergistic effect realized the inhibition of AS progression in mice.

## Supplementary Information

**Additional file 1**: **Figure S1**. Fluorescence images observed the successful isolation of macrophage membranes. The RAW264.7 cells were co-stained with DAPI (blue) and DiI (red), respectively (scale bar: 50 μm). **Figure S2**. (A)The protein bands of Lips-SHP1i (I), macrophages (II), macrophage membranes (III) and MM@Lips-SHP1i (IV) determined by SDS-PAGE electrophoresis assay. (B) SRA expression of Lips-SHP1i (I), macrophages (II), macrophage membranes (III) and MM@Lips-SHP1i (IV) using western blotting. **Figure S3**. CD80 and CD206 expressions in macrophages, M1 macrophages and M2 macrophages determined by CLSM. CD80 expression was presented with red, CD206 with green and nucleus with blue. Scale bar = 20 μm. **Figure S4**. Representative pictures of Oil Red O staining of intracellular lipid droplets in RAW264.7 macrophages after different treatments (scale bar: 50 μm). **Figure S5**. (A) The levels of pro-inflammatory cytokines, including TNF-α, IL-6 and IFN-γ in RAW264.7 supernatant after liposome treatment. (B) Fluorescence images and quantitative analysis observed the effect of different nanoparticles on ROS generation in RAW264.7 cells (scale bar: 50 μm; ***P<0.001). **Figure S6**. Body weight change curves in various groups during treatment. **Figure S7**. Representative photographs and quantitative analysis of aorta root sections stained by CD31 antibody and KI67 antibody (n = 3, scale bar: 200 μm, **P<0.01, ***P<0.001). **Figure S8**. Representative immunofluorescence images and quantitative analysis of aorta root sections stained for cleaved caspase-3 to assess apoptotic cells in lesions. The percentage of cleaved caspase-3+ area was calculated by the total atherosclerotic plaque area in serial sections (scale bar: 100 μm, **P<0.01, ***P<0.001).

## Data Availability

All data generated or analyzed during this study are included in this published article and its Additional file 1.
