# Supplementary material for: Pro-efferocytic macrophage membrane biomimetic nanoparticles for the synergistic treatment of atherosclerosis via competition effect
Source: J Nanobiotechnology. 2022 Dec 1;20:506. doi: 10.1186/s12951-022-01720-2 (PMC9714205; doi:10.1186/s12951-022-01720-2)
Supplement: Supplementary file 1 — Additional file 1: Figure S1. Fluorescence images observed the successful isolation of macrophage membranes. The RAW264.7 cells were co-stained with DAPI (blue) and DiI (red), respectively (scale bar: 50 μm). Figure S2. (A)The protein bands of Lips-SHP1i (I), macrophages (II), macrophage membranes (III) and MM@Lips-SHP1i (IV) determined by SDS-PAGE electrophoresis assay. (B) SRA expression of Lips-SHP1i (I), macrophages (II), macrophage membranes (III) and MM@Lips-SHP1i (IV) using western blotting. Figure S3. CD80 and CD206 expressions in macrophages, M1 macrophages and M2 macrophages determined by CLSM. CD80 expression was presented with red, CD206 with green and nucleus with blue. Scale bar = 20 μm. Figure S4. Representative pictures of Oil Red O staining of intracellular lipid droplets in RAW264.7 macrophages after different treatments (scale bar: 50 μm). Figure S5. (A) The levels of pro-inflammatory cytokines, including TNF-α, IL-6 and IFN-γ in RAW264.7 supernatant after liposome treatment. (B) Fluorescence images and quantitative analysis observed the effect of different nanoparticles on ROS generation in RAW264.7 cells (scale bar: 50 μm; ***P<0.001). Figure S6. Body weight change curves in various groups during treatment. Figure S7. Representative photographs and quantitative analysis of aorta root sections stained by CD31 antibody and KI67 antibody (n = 3, scale bar: 200 μm, **P<0.01, ***P<0.001). Figure S8. Representative immunofluorescence images and quantitative analysis of aorta root sections stained for cleaved caspase-3 to assess apoptotic cells in lesions. The percentage of cleaved caspase-3+ area was calculated by the total atherosclerotic plaque area in serial sections (scale bar: 100 μm, **P<0.01, ***P<0.001). [file 12951_2022_1720_MOESM1_ESM.docx]

**Supplementary Information**


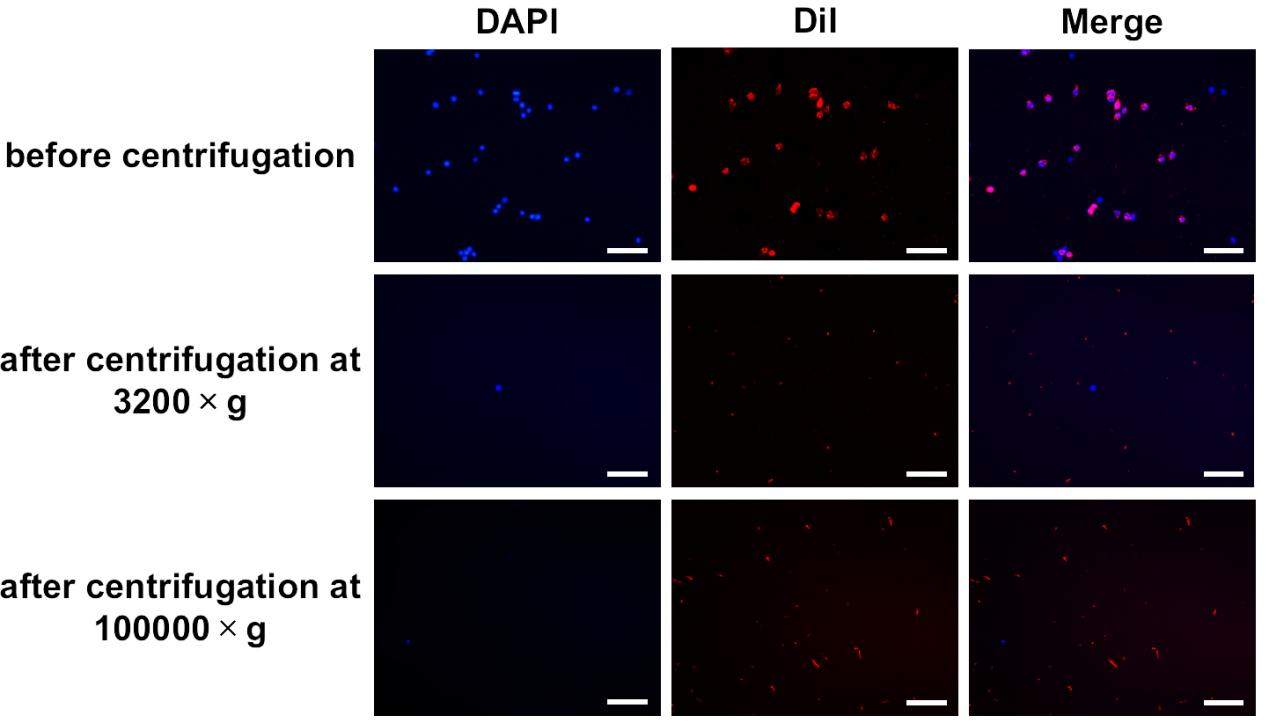


**Figure S1. Fluorescence images observed the successful isolation of macrophage membranes. The RAW264.7 cells were co-stained with DAPI (blue) and DiI (red), respectively (scale bar: 50 μm).**


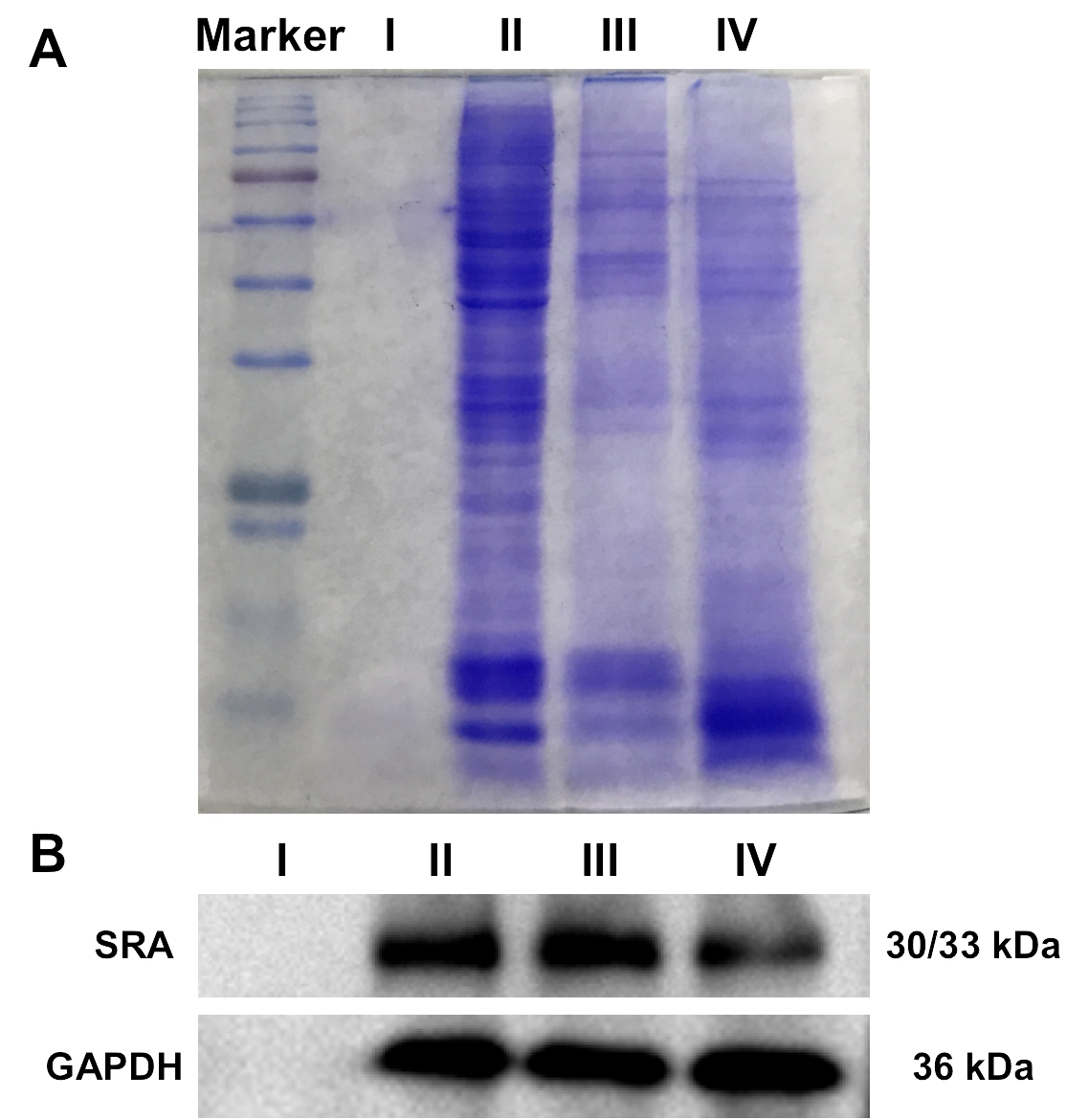


**Figure S2. (A)The protein bands of Lips-SHP1i (I), macrophages (II), macrophage membranes (III) and MM@Lips-SHP1i (IV) determined by SDS-PAGE electrophoresis assay. (B) SRA expression of Lips-SHP1i (I), macrophages (II), macrophage membranes (III) and MM@Lips-SHP1i (IV) using western blotting.**

**
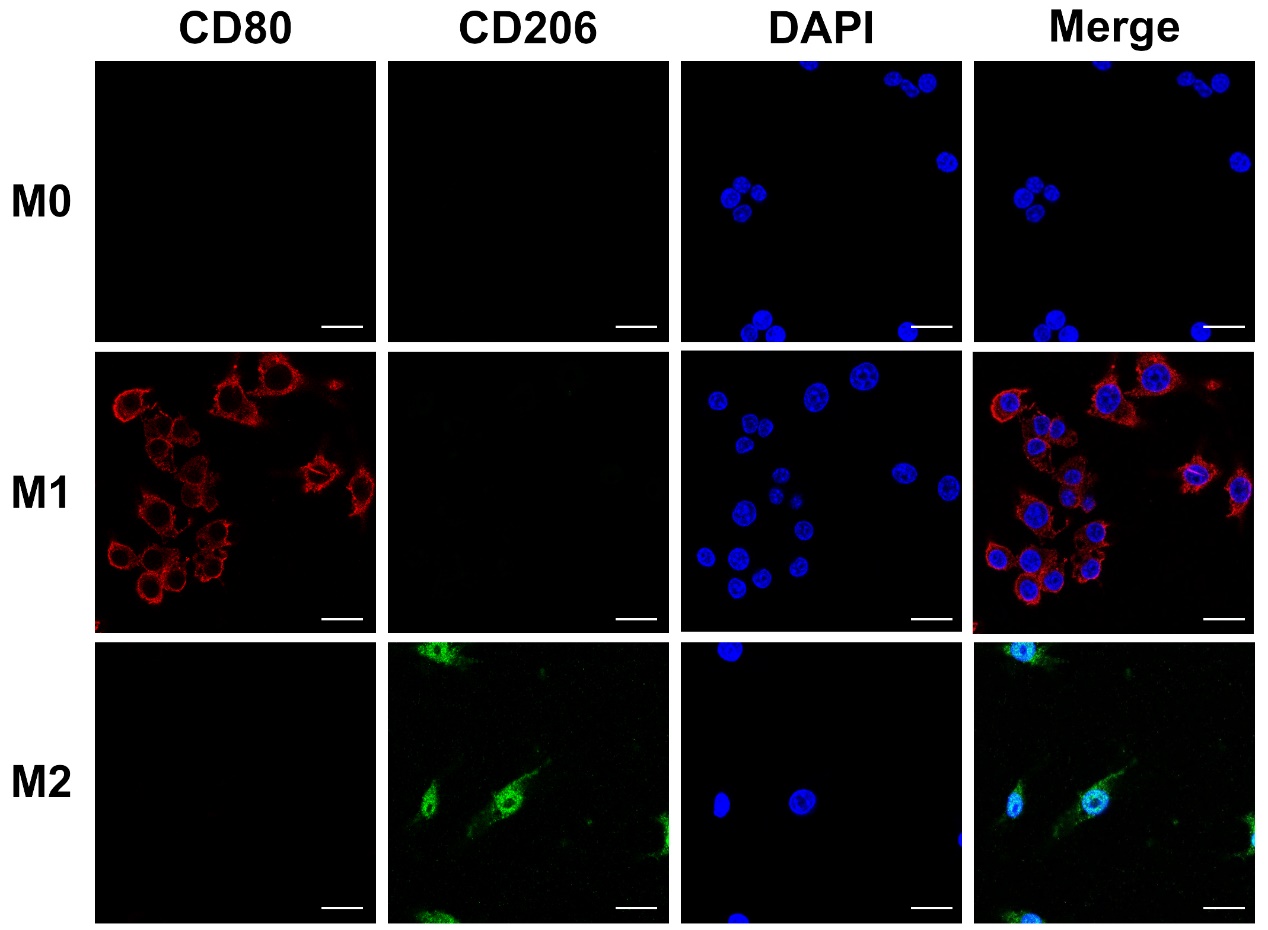
**

**Figure S3. CD80 and CD206 expressions in macrophages, M1 macrophages and M2 macrophages determined by CLSM. CD80 expression was presented with red, CD206 with green and nucleus with blue. Scale bar = 20 μm.**


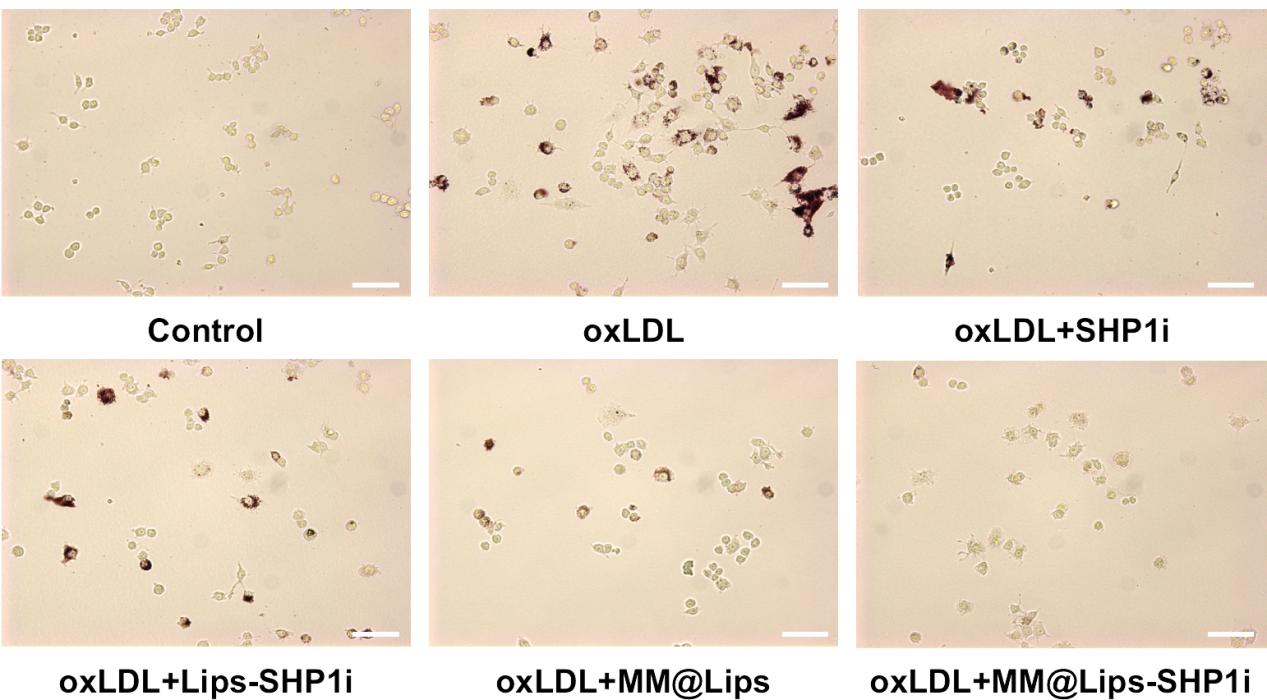


**Figure S4.** **Representative pictures of Oil Red O staining of intracellular lipid droplets in RAW264.7 macrophages after different treatments. Scale bar = 50 μm.**


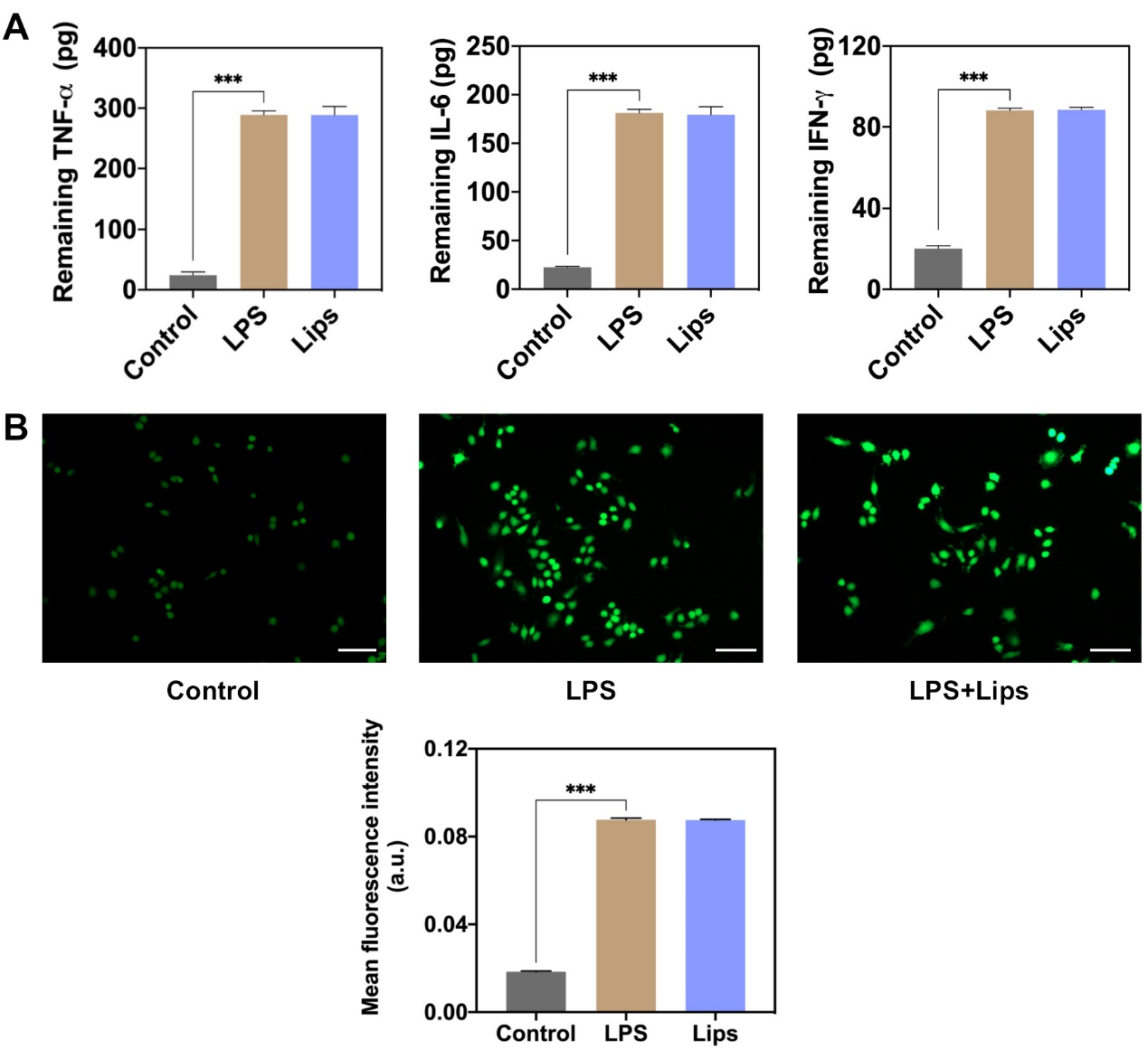


**Figure S5** **(A) The levels of pro-inflammatory cytokines, including TNF-α, IL-6 and IFN-γ in RAW264.7 supernatant after liposome treatment. (B) Fluorescence images and quantitative analysis observed the effect of different nanoparticles on ROS generation in RAW264.7 cells (scale bar: 50 μm; ****P*<0.001).**


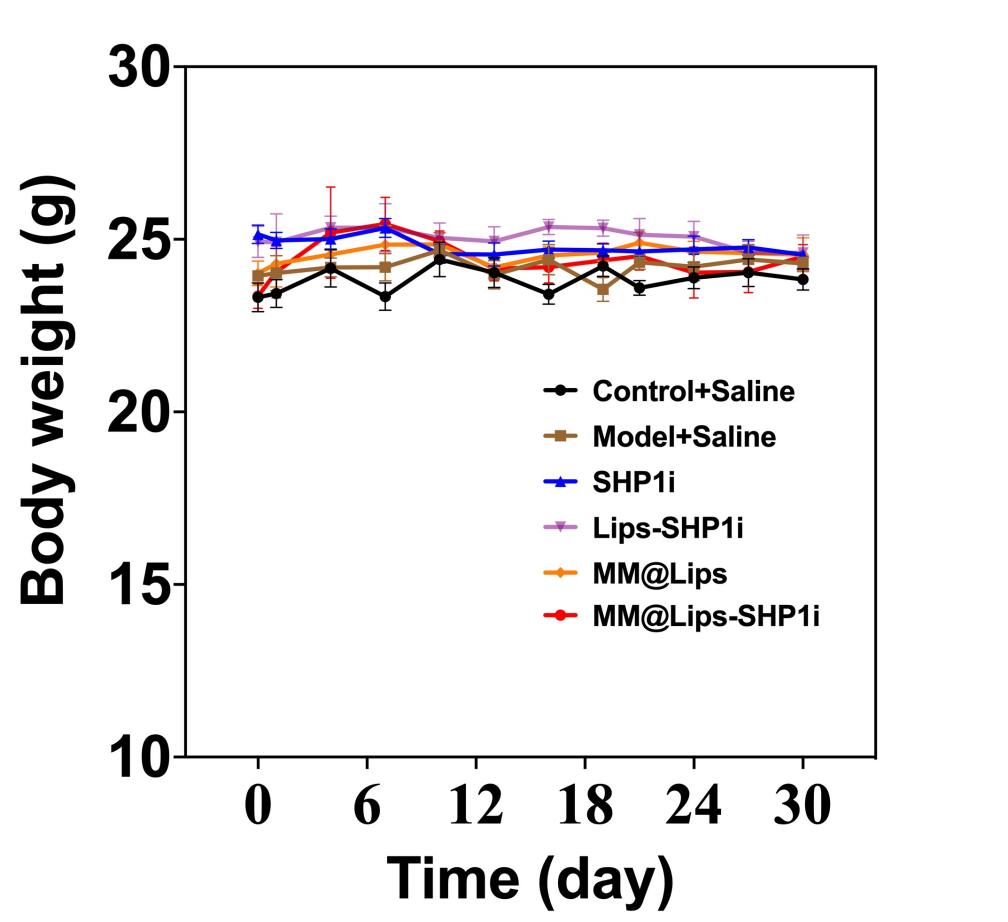


**Figure S6.** **Body weight change curves in various groups during treatment.**


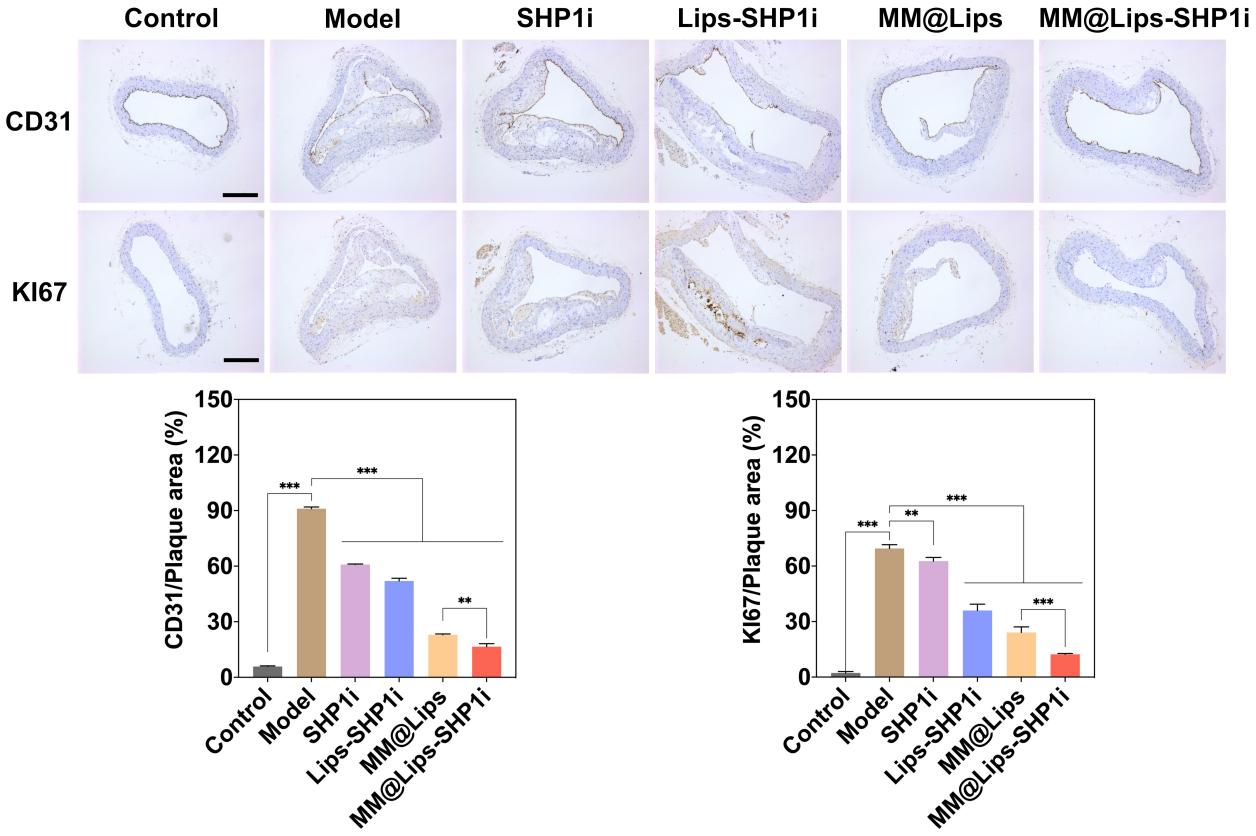


**Figure S7. Representative photographs and quantitative analysis of aorta root sections stained by CD31 antibody and KI67 antibody (n = 3, scale bar: 200 μm, ***P*<0.01, ****P*<0.001).**


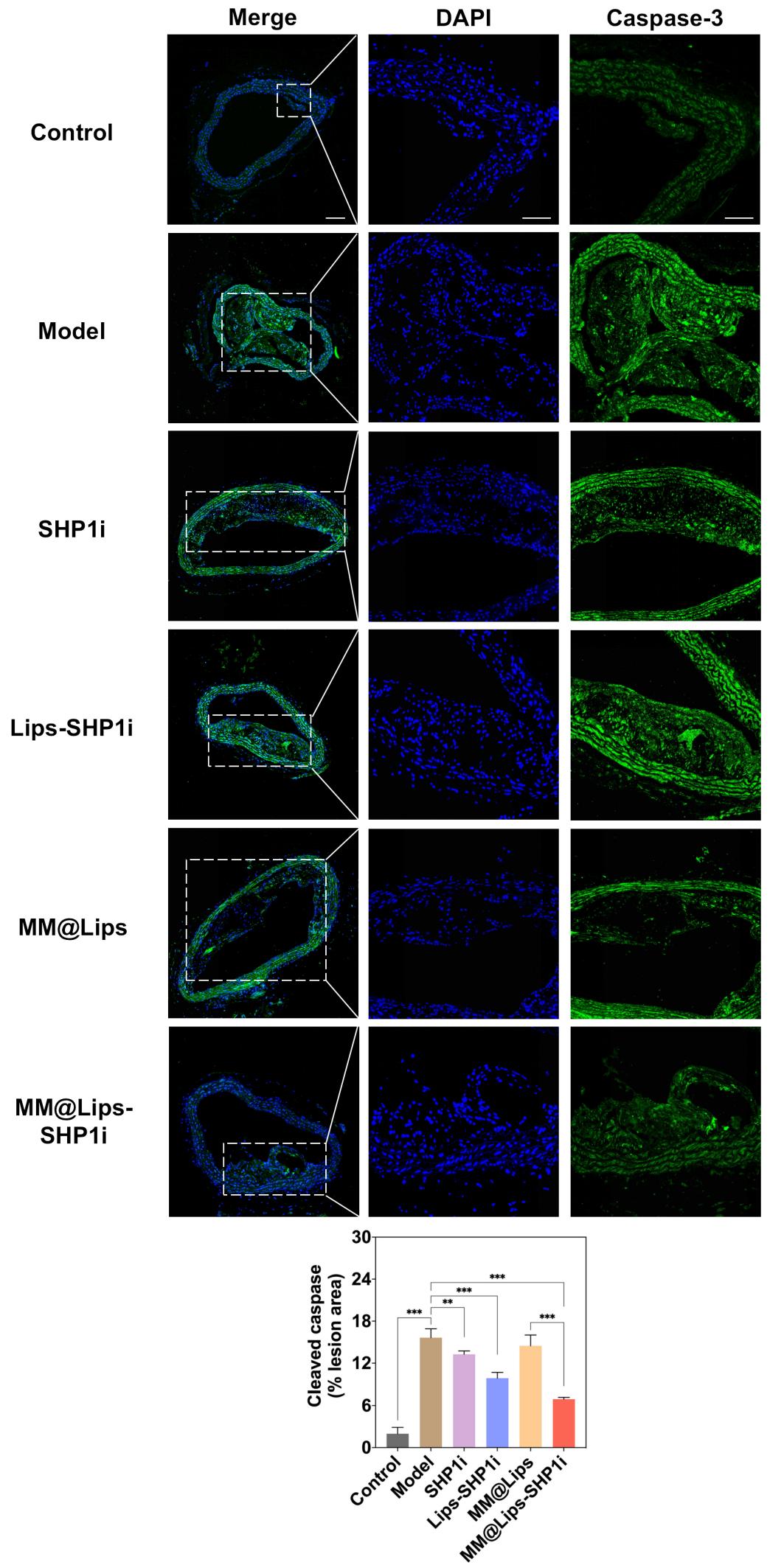


**Figure S8. Representative immunofluorescence images and quantitative analysis of aorta root sections stained for cleaved caspase-3 to assess apoptotic cells in lesions. The percentage of cleaved caspase-3^+^ area was calculated by the total atherosclerotic plaque area in serial sections (scale bar: 100 μm, ***P*<0.01, ****P*<0.001).**
